# Chick fetal organ spheroids as a model to study development and disease

**DOI:** 10.1186/s12860-021-00374-6

**Published:** 2021-07-05

**Authors:** Soran Dakhel, Wayne I. L. Davies, Justin V. Joseph, Tushar Tomar, Silvia Remeseiro, Lena Gunhaga

**Affiliations:** 1 Umeå Centre for Molecular Medicine, Umeå University, 901 87 Umeå, Sweden; 2 Wallenberg Centre for Molecular Medicine, Umeå University, 901 87 Umeå, Sweden; 3 PamGene International B.V, Wolvenhoek 10, 5211 HH ‘s-Hertogenbosch, The Netherlands

**Keywords:** Fetal organ spheroids, Chick, 3D cell culture, Development, Cancer, Invasion

## Abstract

**Background:**

Organ culture models have been used over the past few decades to study development and disease. The in vitro three-dimensional (3D) culture system of organoids is well known, however, these 3D systems are both costly and difficult to culture and maintain. As such, less expensive, faster and less complex methods to maintain 3D cell culture models would complement the use of organoids. Chick embryos have been used as a model to study human biology for centuries, with many fundamental discoveries as a result. These include cell type induction, cell competence, plasticity and contact inhibition, which indicates the relevance of using chick embryos when studying developmental biology and disease mechanisms.

**Results:**

Here, we present an updated protocol that enables time efficient, cost effective and long-term expansion of fetal organ spheroids (FOSs) from chick embryos. Utilizing this protocol, we generated FOSs in an anchorage-independent growth pattern from seven different organs, including brain, lung, heart, liver, stomach, intestine and epidermis. These three-dimensional (3D) structures recapitulate many cellular and structural aspects of their in vivo counterpart organs and serve as a useful developmental model. In addition, we show a functional application of FOSs to analyze cell-cell interaction and cell invasion patterns as observed in cancer.

**Conclusion:**

The establishment of a broad ranging and highly effective method to generate FOSs from different organs was successful in terms of the formation of healthy, proliferating 3D organ spheroids that exhibited organ-like characteristics. Potential applications of chick FOSs are their use in studies of cell-to-cell contact, cell fusion and tumor invasion under defined conditions. Future studies will reveal whether chick FOSs also can be applicable in scientific areas such as viral infections, drug screening, cancer diagnostics and/or tissue engineering.

**Supplementary Information:**

The online version contains supplementary material available at 10.1186/s12860-021-00374-6.

## Background

Models that recapitulate paradigms of organ development in vitro have profound implications for understanding the nature of organ formation and disease. Avian embryos, in particular the chick, have been instrumental to the field of developmental biology, and have also made significant contributions to studies in; cell biology, neuroscience, virology, immunology and cancer biology [1–3]. Many of the major concepts in developmental biology, such as those of cell type induction and competence, plasticity and contact inhibition, are an outcome of work using chick as a model system, with results that have major relevance to other areas of biology [2]. In particular, the chick embryo has many similarities to the human embryo at molecular, cellular and anatomical levels, thus the chick is a valid and useful model to study human gene and cellular function. Specifically, orthologous genes are conserved between the chick (*Gallus gallus*) and human (*Homo sapiens*), with a high level of synteny present between their respective genomes [4]. In addition, the genetic mutation rate in humans more closely resembles that of the chicken versus the very fast rate of nucleotide substitution found in rodents [2, 5]. Also, with the introduction of state-of-the-art techniques such as next-generation sequencing and genome editing, the chick offers renewed relevance to comparative genomics and studies of the regulatory non-coding genome that are associated with vertebrate development, biology and evolution [6]. Finally, the chick model offers substantial economic and practical advantages, such as low cost, the availability of large quantities of fertilized eggs throughout the year, and robust and easily accessible embryos.

Recent technical advances have led to the development of novel in vitro three-dimensional (3D) culture systems called organoids [7, 8]. Organoids are most often generated from embryonic stem cells (ESCs), induced pluripotent stem cells (iPSCs) or tissue-resident stem/progenitor cells [9], which spontaneously self-organize into properly differentiated functional cell types that recapitulate at least some functions of the organ [10, 11]. In many cases, organoids aim to model human development and disease mechanisms (reviewed in [12, 13]). However, organoid models are generally both expensive and difficult to culture and maintain. Furthermore, most organoids are dependent on a selective matrix for its growth, and hence present an anchorage-dependent growth pattern that is somewhat dissimilar to the in vivo cellular milieu. An inexpensive, time efficient and simplified model to maintain 3D cell culture spheroids, in an anchorage independent growth system, would at least partly substitute and complement the use of organoid cultures.

Considering the advantages of chick as a model system, and the relevance and interest in generating in vitro organ cultures, we have generated an in vitro 3D chick organ culture assay based on fetal organ spheroids (FOSs). We present the generation and characterization of FOSs from seven different organs harvested from chick embryos, namely brain, lung, heart, liver, stomach, intestine and epidermis. The growth kinetics of these FOSs were studied, as well as the expression of representative organ and cell markers, the latter of which were compared to corresponding in vivo organs at relevant developmental time-points. Different cell types and their distribution within the FOSs were validated by fluorescence and electron microscopy (EM). Together, our data indicate that the generated in vitro 3D FOSs exhibit in vivo organ-like characteristics. Furthermore, a confrontation assay between FOSs and cancer cell spheres indicates a cell-to-cell preference depending on tumor types and target organs. Thus, a potential application of chick FOSs are their use in studies of cell-to-cell contact, cell fusion and tumor invasion under defined conditions.

## Methods

### Chick embryos

Fertilized white Lohman and Bovan chicken eggs (*Gallus gallus*) were obtained from Strömbäcks Ägg, Vännäs, Sweden, and incubated in a 38 °C humidified egg incubator. The embryos were harvested at embryonic day (E) 12 and E14, and staged according to the protocol of Hamburger and Hamilton [14]. Embryos were maintained on ice in phosphate-buffered saline (PBS, pH 7.4) prior to organ dissection. The use of fertilized chick eggs prior to E14 does not require ethical permission. Experiments using chick embryos at E14 was approved by the Institutional Animal Care and Use Committee of Umeå University, Sweden (Dnr A14–17).

### Fetal organ processing

Five samples of each organ, brain, heart, liver, stomach and epidermis from the top of the head were extracted from E12 embryos, and lung and intestine from E14 embryos. All tissues were kept in ice cold PBS until processed further. Organs were transferred to 500 μl of cold PBS and minced finely with sterile scissors. Organ suspensions were centrifuged at 900 rpm for 10 min at 4 °C, and the resultant cell pellets were incubated in 4 ml of Accutase (Sigma-Aldrich, A6964) at 37 °C. The incubation time with Accutase was adjusted according to the consistencies of the organs (Fig. S1d). After incubation, 6 ml of RPMI medium (Gibco), containing 10% fetal bovine serum (FBS; Life Technologies) and 1% penicillin-streptomycin (PS; Gibco), was added to the cell suspension. The cell suspension was pipetted repeatedly until the cells were dissociated, followed by centrifugation at 900 rpm for 10 min at RT. The obtained pellet was resuspended in 10 ml fresh RPMI medium and passed through a 70 μm (not larger) cell strainer (Corning CLS431751). The cells were counted using Countess II FL Automated Cell Counter (Thermofisher) to check the percentage of viable cells. The fetal organ processing is summarized in Figure S1.

### Fetal organ spheroid (FOS) culture and imaging

Ten million dissociated organ cells were seeded in a 25 cm^2^ flask coated with 1.5% agarose (Fisher Bioreagents) in a total volume of 7 ml RPMI medium (Gibco) containing 10% FBS (Life Technologies) and 1% PS (Gibco), and cultured. To prevent the organ spheroids from clustering, the contents in the flasks were gently pipetted for the first 2 days after cell seeding and thereafter when changing medium. The medium was changed every 3–4 days by transferring the spheroids to a 15 ml tube followed by centrifugation at 500 rpm for 5 min. Then 1 ml of media containing the spheroids were seeded in 6 ml of fresh media. The FOSs were harvested for further use at Day (D) 4, D8 and D12. FOSs were examined by Live cell microscope (Eclipse Ti-E, Nikon), and images were captured using an Andor DU-885 X-3326 camera and Nikon NIS-Elements software.

### RNA extraction, cDNA synthesis and RT-PCR analysis

Total RNA was derived from pooled D8 FOSs, and from dissected chick organs at E12 (brain, heart, liver, stomach, and epidermis) (*n* = 5) or at E14 (lung and intestine) (*n* = 5). RNA was extracted using RNeasy Mini kit (Qiagen), and the RNA concentrations were assessed by a Nanodrop spectrophotometer (Thermo Scientific). To avoid DNA contamination samples were treated with RNAase-free DNase I (Invitrogen) before complementary (c) DNA (cDNA) was synthesized using Superscript IV RT (Invitrogen). Table S2 includes information of primer sequences, amplicon size and annealing temperatures for running the reverse transcription (RT)-PCR for the following genes; G*lial fibrillary acidic protein* (*GFAP*), *Surfactant protein C* (*SP-C*), *Cardiac muscle troponin T2 (cTnT or TNNT2), Albumin (Alb), Homeobox protein BarH-like 1 (Barx1*)*, Sucrose-isomaltase* (*SI*), *Epidermal differentiation protein starting with a MTF motif and rich in histidine* (*EDMTFH*), *Vimentin* (*Vim*) and *glyceraldehyde-3-phosphate dehydrogenase* (*GAPDH*, used as a reference gene). PCRs were conducted using MyTaq Red Mix (Bioline) according to the manufacturer’s instructions and by applying the following amplification conditions: 95 °C 5 min, 35 cycles [95 °C 30 s, T°C 30 s, 72 °C 1.5 min], 72 °C 5 min. See Table S2 for annealing temperatures (T°C).

### Immunocytochemistry and imaging

For immunocytochemistry (ICC), spheroids were fixed in 4% paraformaldehyde (PFA, Fisher Scientific) for 1 h at 4 °C, transferred to 25% sucrose for 30 min at 4 °C, embedded in agarose (Fisher Bioreagents) and thereafter in NEG-50 frozen section medium (Thermo Fisher Scientific). Organs were fixed in 4% PFA for 1 h at 4°C, transferred to 25% sucrose and embedded in NEG-50. All tissue blocks were stored at − 80 °C until cryo-sectioned at 10 μm on consecutive slides. ICC was performed using standard protocols [15]. Briefly, sections were blocked with 10% FBS prior to primary antibody incubation at 4 °C overnight (ON). Primary antibodies were used as follows: anti-E-cadherin (mouse, 1:50; DSHB), anti-HuC/D (mouse, 1:200; Molecular Probes), anti-Nkx2.1 (mouse, 1:500; BIOPAT Immunotechnologies), anti-cleaved Caspase 3 (rabbit, 1:1000; Cell Signaling Technology), anti-phospho-Histone H3 (rabbit, 1:500; Merck Millipore), anti-GFAP (rabbit, 1:500; gift from Anna Överby Wernstedt, Umeå University, Sweden), anti-Sox2 (rabbit, 1:800; gift from Thomas Edlund, Umeå University, Sweden), anti-SP-C (rabbit, 1:200; Santa Cruz), and anti-Somatostatin (rat, 1:100; gift from Helena Edlund, Umeå University, Sweden). Secondary antibodies used were; anti-mouse Alexa Fluor 488 and 594 (1:400, Invitrogen), anti-rabbit Cy3 (1:400, Jackson Immuno Research), and anti-rat Alexa Fluor 594 (1:400, gift from Helena Edlund, Umeå University, Sweden). Nuclei were detected using 4′,6-diamidino-2-phenylindole, dihydrochloride (DAPI, 1:400; Sigma-Aldrich). If needed, TrueBlack (Biotium) was added to minimize the presence of autofluorescence. Sections were mounted with fluorescence mounting medium (Agilent Technologies). The stained sections were examined by fluorescence microscopy (Nikon Eclipse, E800), with images being captured using a Nikon DS-Ri1 digital camera and Nikon NIS-Elements F v4.6 software.

### Electron microscopy

#### Scanning electron microscopy (SEM)

The FOS samples were fixed with 2.5% glutaraldehyde (GA) in 0.1 M sodium cacodylate buffer and sedimented for 1 h on glass coverslips. Subsequently, the samples were dehydrated in series of graded ethanol, critical point dried and coated with 5 nm platinum. The morphology of the samples was analyzed by field-emission scanning electron microscopy (FESEM; Carl Zeiss Merlin) using a secondary electron detector at an accelerating voltage of 4.00 kV and a probe current of 100 pA.

#### Transmission electron microscopy (TEM)

The TEM protocol used here essentially followed the Tokuyasu technique [16]. Spheroids were fixed in 2% PFA/ 0.2% GA (Taab Laboratories Equipment Ltd) at 4 °C ON, and then replaced with 1% PFA for 1 h at 4 °C. The fixed spheroids were washed with PBS, followed by incubation in 0.1% glycine (Merck) in PBS for 10 min at RT. The solution was then replaced with 12% Gelatine (Dr. Oetker) in 0.1 M PB (pH 7.4) and incubated at 37 °C for 30 min, followed by the same procedure, but with a 10 min incubation at 37 °C and then 30 min on ice. Gelatin embedded spheroids were transferred to 2.3 M sucrose ON at 4 °C. Approximately 0.5 mm^2^ spheroids were mounted on metal pins with 2.3 M sucrose and instantly frozen in liquid nitrogen. 70 nm Ultra microtome (Leica) sections were placed on parallel copper grids (Taab Laboratories Equipment Ltd) with formvar film and the carbon layer of ~ 3 nm. Sections were incubated in PBS for 5 min at RT, then for 20 min at 37 °C, before washing in PBS and then sterile H_2_O. Contrasting was performed by incubation with 0.4% uranyl acetate in 2% methyl cellulose (pH 4), on ice for 5 min. The grids were looped out using remanium wire loops, and imaged on a transmission electron microscope (JEOL JEM-1230) equipped with a Gatan Orius 830 2kx2k CCD camera (JEOL) using Digital micrograph software.

### Cell culture and tumor sphere formation

Two human cancer cell lines that stably express green fluorescent protein (GFP) were used, namely human glioblastoma U251-*GFP* and human lung cancer A549-*GFP* [17]. The cell lines were cultured in RPMI medium (Gibco) supplemented with 10% FBS (Life Technologies), and 1% PS (Gibco), and maintained below 15 passages. 3D spheres were generated by culturing the A549-*GFP* and U251-*GFP* cells in flasks coated with 1.5% agarose at a density of 2 million cells per flask, with medium being changed every 3–4 days. At culture day 8, tumor spheres of similar sizes as the FOS were used in the confrontation assays. The A549 cell line was initially purchased from the American Type Culture Collection (ATCC), and the U251 MG (formerly U-373 MG) originated from the European Collection of Authenticated Cell Cultures (ECACC). Mycoplasma tests (Eurofins GATC Biotech) confirmed that the cancer cells were free from mycoplasma infection, and the identity of the cancer cell lines was verified by STR profiling (Microsynth).

### FOS and tumor sphere confrontation assay and imaging

Day 8 cultured tumor spheres from either A549-*GFP* or U251-*GFP* cells were individually used in confrontation assays with either D8 FOSs from brain (FBS) or lung (FLuS). Using pipettes, one FOS and one tumor sphere were plated together in 8 well chamber coverslip (Ibidi) coated with 1.5% agarose. The chamber was gently moved back and forth, while still monitoring under a microscope, to establish a confrontation between the FOS and the tumor sphere. 4 times during the 10 days culture period 50% media were replenished. Once a day for up to 10 days, potential fusion and invasion were monitored with bright field and fluorescence microscopy, respectively, as described above. At Day 10, confronted spheroids/spheres were fixed in 4% PFA for 30 min at 4 °C, washed in PBS and incubated overnight in DAPI (1:400; Sigma-Aldrich) at 4 °C. The spheroids/spheres were mounted on slides in glycergel aqueous mounting medium (Agilent Technologies) without coverslips, and representative samples were subjected to confocal microscopy using a fully automated Nikon A1 confocal microscope paired with NIS-Elements C software. Exposure times were chosen to maximize visualization of the tumor-FOS interface. Images were captured at an interval of 2 μm and incorporated in 3D z-stacks. Dynamic scanning movies were generated offline using NIS-Elements BR-5.02 software. iMovie (Mac) was used to edit the movies.

### Statistical analyses

The perimeter of each FOS at D4, D8 and D12 were quantified using NIH Image J software [18] from 10x bright field microphotographs. A freehand measurement tool was used after setting the scale at 10x magnification (107 pixels = 100 μm). For each organ type and specified day, a minimum of 75–100 spheroids were analyzed and measured. Significance was determined by applying Student’s two-tailed paired *t*-test, and *P*-values of *P < 0.05 were accepted as being statistically significant. All statistical analyses were performed using GraphPad PRISM 8.1.2.

## Results

### Generation of fetal organ spheroids (FOSs)

An overview of the steps involved in the generation of chick fetal organ spheroids (FOSs) is shown in Figure S1. Brain, heart, liver, stomach, and epidermis from the top of the head, were collected from embryonic day (E) 12, and E14 embryos were used to harvest lung and intestine, stages when respective organs were clearly defined (Figs. S1a, b). The dissected organs were maintained in phosphate-buffered saline (PBS) on ice until further processing, including mincing and enzymatic dissociation of the tissue (Fig. S1c). The duration of enzymatic dissociation ranged from 5 to 20 min depending on organ structure (Fig. S1d). After analyzing the percentage of viable cells from each organ, single cells were seeded at a density of 10 × 10^6^ cells/flask in agarose-coated flasks in supplemented RPMI medium. Over time, seeded cells clustered together, with visible spheroid-like structures already from day one (Fig. S2).

### Morphological characterization and growth of FOSs

**Fig. 1:**
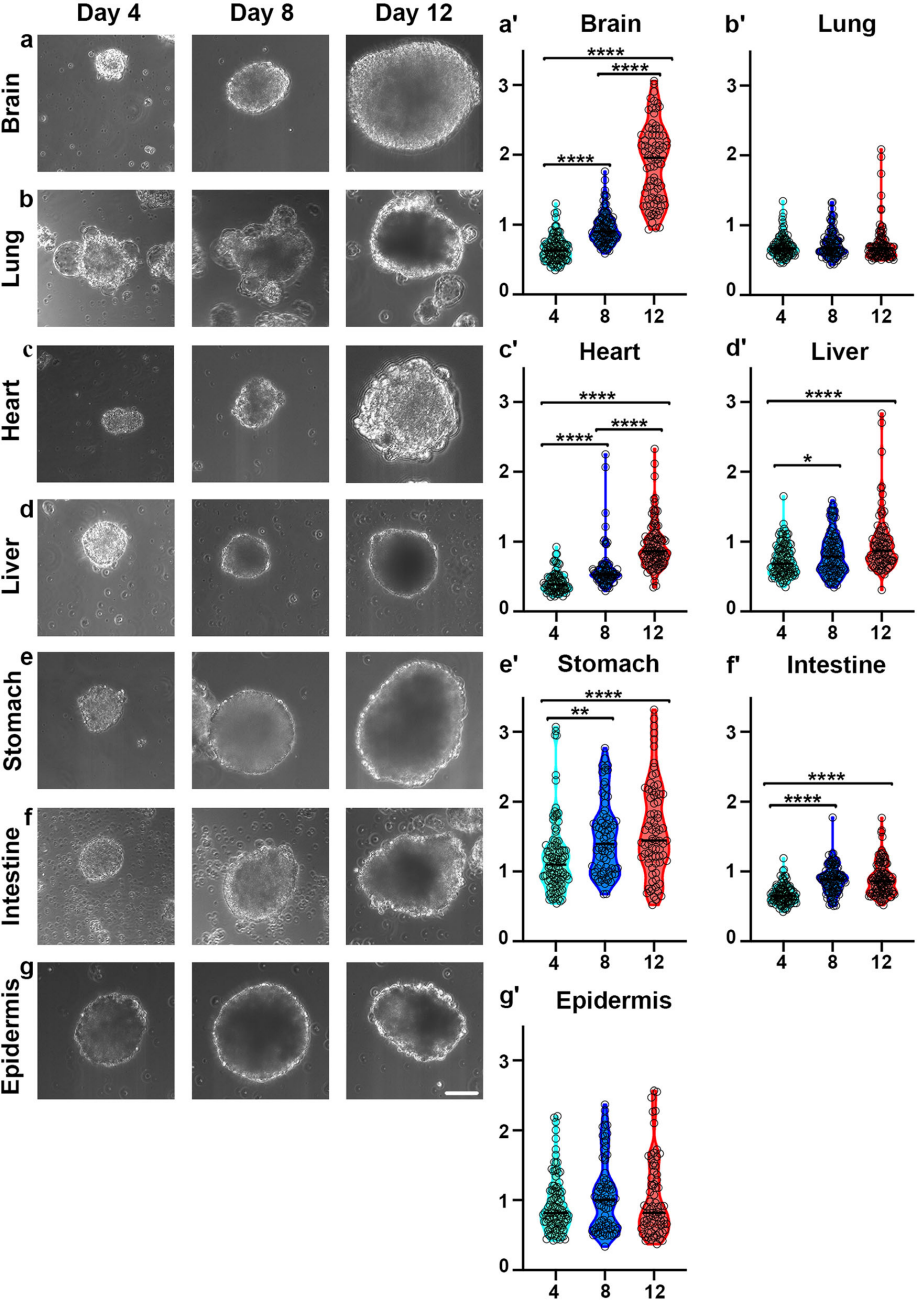
Morphology and growth of seven different FOSs at day 4, 8 and 12. **a**, **c**-**f** An increase in perimeter of the brain, heart, liver, stomach and intestinal spheroids was observed. **b**, **g** No apparent growth was observed in the lung and epidermis spheroids between day 4 and 12. **a**′-**g**′ Day 4 spheroids in light green, day 8 spheroids in blue and day 12 spheroids in red. The x-axes indicate days in culture, and the y-axes indicate the perimeter of the fetal organ spheroids in mm. *P < 0.05, **P < 0.01, ****P < 0.0001. Scale bar: 100 μm (**a**-**g**)

FOSs (presented from anterior to posterior) derived from brain, lung, heart, liver, stomach, intestine and epidermis, consistently displayed a 3D spheroid-like morphology with a general increase in size over time from day 4–12 (Fig. 1). Different FOSs exhibited some morphological variation depending on the organ of origin; for example, FOSs generated from brain mostly exhibited a spherical morphology (Fig. 1a), whereas stomach FOSs were more ellipsoidal (Fig. 1e). Smaller FOSs generated from lung, heart, intestine and epidermis were all irregular in shape, whereas the larger spheroids were more spherical (Fig. 1b, c, f, g). Interestingly, from day 3–4 of culture, lung FOSs exhibited the presence of differently sized globular sac-like structures adjacent to and around the spheroids, and these sac-like structures remained intact for several days in culture (Fig. 1b).

Differences in growth kinetics of various FOSs were observed (Table S1). Brain spheroids showed the most rapid increase in perimeter over time, with a 2.9-fold increase from day 4–12, when reaching 1.88 ± 0.06 mm (Fig. 1a′). Long-term culture up to a month of the fetal brain spheroids did, however, not increase the size further (data not shown). The perimeter of heart spheroids increased 2.2-fold from day 4–12, and reached 0.94 ± 0.03 mm at day 12 (Fig. 1c′). Meanwhile, liver, stomach and intestinal spheroids all showed a 1.3-fold increase of the perimeter (Fig. 1d′-f′), whereas lung and epidermal spheroids did not grow significantly during the culture time (Fig. 1b′, g′). Notably, stomach and epidermal spheroids were already 1.21 ± 0.06 mm and 0.94 ± 0.04 mm in perimeter, respectively, at day 4 (Fig. 1e′, g′), which might explain lack of or slower growth between day 4 and 12. Thus, the perimeter and morphological appearance of the spheroids varied in some extent from one organ type to another.

**Fig. 2:**
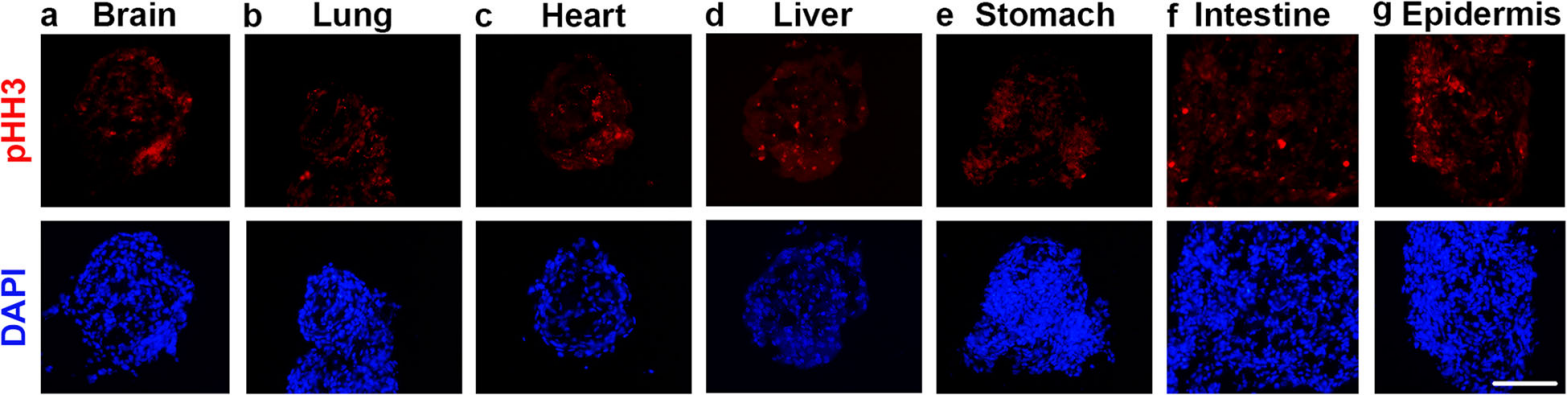
Expression of phosphorylated Histone H3 (pHH3) detected by immunocytochemistry in day 8 FOSs. **a**-**g** pHH3^+^ cells, indicative of cell proliferation, were observed in all FOSs derived from seven different organs (*n* = 5 for all FOSs). DAPI indicates nuclei. Scale bar: 100 μm

The increase in size of the FOSs implicated ongoing proliferation. To identify the presence of mitotic cells in FOSs, the proliferative marker phosphorylated (p) histone H3 (pHH3), which labels proliferating cells in late G2 and M phases [19], was analyzed by immunocytochemistry. pHH3^+^ proliferative cells were observed in all FOSs at all time points analyzed, with no clear difference in proliferation rate between spheroids or throughout culture period (Fig. 2a-g and data not shown). Even if lung and epidermal spheroids did not show an apparent increase in size over time between days 4 and 12 in culture, the presence of pHH3^+^ cells indicates that some degree of cell renewal is also occurring in these spheroids. Guided by the growth and proliferation results, all subsequent studies were performed on FOSs from day 8 of culture to represent a median time point.

**Fig. 3:**
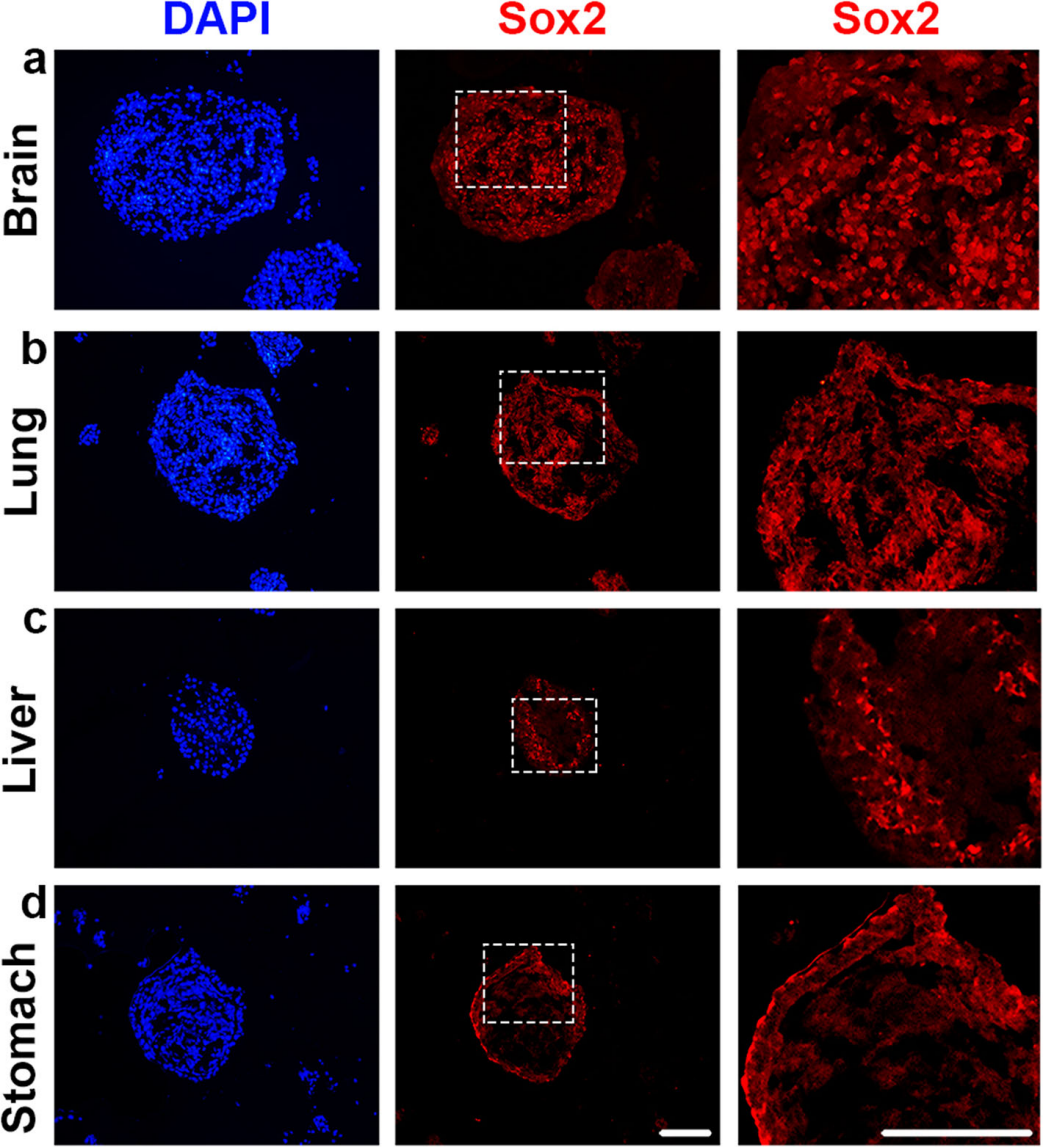
Sox2 expression detected by immunocytochemistry in day 8 brain, lung, liver and stomach spheroids. **a**-**b** In the brain and lung FOSs (*n* = 7 for both), Sox2 expression was detected throughout the spheroidal mass. **c**-**d** In the liver and stomach FOSs (*n* = 7 for both), Sox2 expression was primarily detected in the periphery of the spheroid. The right panels indicate a magnified view of the area highlighted by white dotted boxes of the middle panels. **a**-**d** DAPI indicates nuclei. Scale bars: 100 μm

Next, we examined whether the pluripotent and progenitor marker SRY-box 2 (Sox2) [20, 21] was expressed in any of the FOSs. By applying immunocytochemistry, scattered Sox2 staining was detected throughout the brain, lung and liver spheroids (Fig. 3a-c), while stomach spheroids showed Sox2 expression primarily in cells lining the surface of the spheroids (Fig. 3d). No expression of Sox2 was observed in heart, intestinal or epidermal spheroids (Figs. S3a-c). In addition, only a few cleaved (c) Caspase3^+^ cells, indicative of apoptotic cells, were detected in the different spheroids (Fig. S4), ruling out a major apoptotic contribution to morphology and growth patterns of the FOSs. Thus, all FOSs appears healthy, contain mitotic cells, and several of the spheroids also express Sox2 indicative of progenitor cells.

### FOSs emulate typical characteristics of in vivo organs

**Fig. 4:**
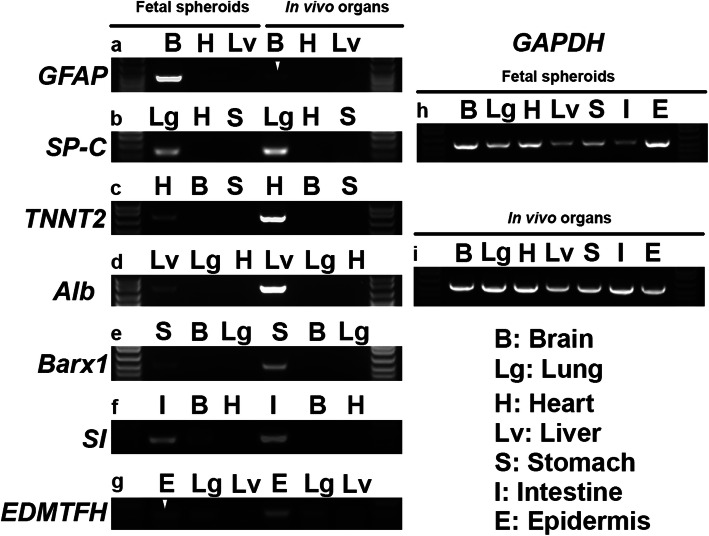
RT-PCR of day 8 FOSs revealed that mRNA expression of typical in vivo organ characteristic genes are enriched in the corresponding FOSs. **a** *GFAP* expression in brain, but not in heart or liver FOSs/organs. **b** *SP-C* expression in lung, but not in heart or stomach FOSs/organs. **c** *TNNT2* expression in heart, but not in brain or stomach FOSs/organs. **d** *Alb* expression in liver, but not in lung or heart FOSs/organs. **e** *Barx1* expression in stomach, but not in brain or lung FOSs/organs. **f** *SI* expression in intestine, low in brain and none in heart FOSs/organs. **g** *EDMTFH* expression in epidermis, and minimal in lung or liver FOSs/organs. **h**, **i** The house-keeping gene *GAPDH* was used as a reference gene. White arrowheads highlight the lower, but consistent observed expression of *GFAP* and *EDMTFH* in brain and epidermal FOSs, respectively. **a**-**i** The first and last lane of each gel image show partial views of the ladder. The presented PCR-gel images are cropped, with full-length gels shown in Fig. S5

To validate the conservation of tissue-like expression profiles of the generated FOSs, the presence of key genes associated with a particular organ type was analyzed by RT-PCR in the FOSs, compared to their corresponding in vivo embryonic organs. In the brain, *GFAP* is expressed in astroglial cell types both in the developing and mature chicken brain [22, 23]. In the lung, the appearance of surfactant-producing alveoli is a hallmark of lung maturation during the transition from the saccular to alveolar stage of lung development [24]. Also in the lung, *Surfactant protein* (*SP*) genes, including *SP-C*, are expressed in a cell-type restricted manner by Clara and/or alveolar type II cells [25]. In the heart of chick and other vertebrates, cardiac troponin T (cTnT), encoded by *TNNT2,* is expressed throughout heart development and at postnatal stages [26]. Albumin, which is exclusively synthesized by the liver, is the most abundant protein in the blood plasma with several physiological roles. In chick, embryonic liver *Albumin* is first detectable at E6.5 and remains expressed during adult stages [27]. In the stomach, the expression of *Homeobox protein BarH-like 1* (*Barx1*) is restricted to the stomach mesenchyme during gut organogenesis [28], and in the mouse, loss of mesenchymal *Barx1* prevents stomach epithelial differentiation [29]. In the intestine’s brush border membrane of mammals and birds, Sucrase-isomaltase (SI), an α-glucosidase enzyme involved in sugar digestion, is expressed [30] . In chicken and other mammals, genes within the epidermal differentiation complex (EDC) are enriched in epidermal cells, one of which is *EDMTFH* in avian skin [31]. The enriched key genes in specific organs mentioned above were examined in both the FOSs and in vivo organs of interest, as well as in two other selected spheroids/organs. As expected, RT-PCR confirmed that similar gene expression profiles typical of the organs of origin, from which the FOSs were derived, were also present in the generated FOSs, but no or low expression in the other two FOSs/organs studied (Fig. 4; Fig. S5). These results indicate that all seven FOSs maintain a good degree of organ-like characteristics for downstream applications.

It is known that mesenchymal-epithelial crosstalk plays a crucial role in organ development [32]. To evaluate whether mesenchymal or mesenchymal-derived cells are present in the FOSs, the expression of *Vimentin* [33] was examined in the seven FOSs using RT-PCR. All FOSs, expressed *Vimentin*, albeit with lower expression in the liver FOSs, which are in line with observed *Vimentin* expression in the corresponding in vivo embryonic organs (Fig. S6). These results suggest that the majority of the established FOSs include cells of mesenchymal character.

**Fig. 5:**
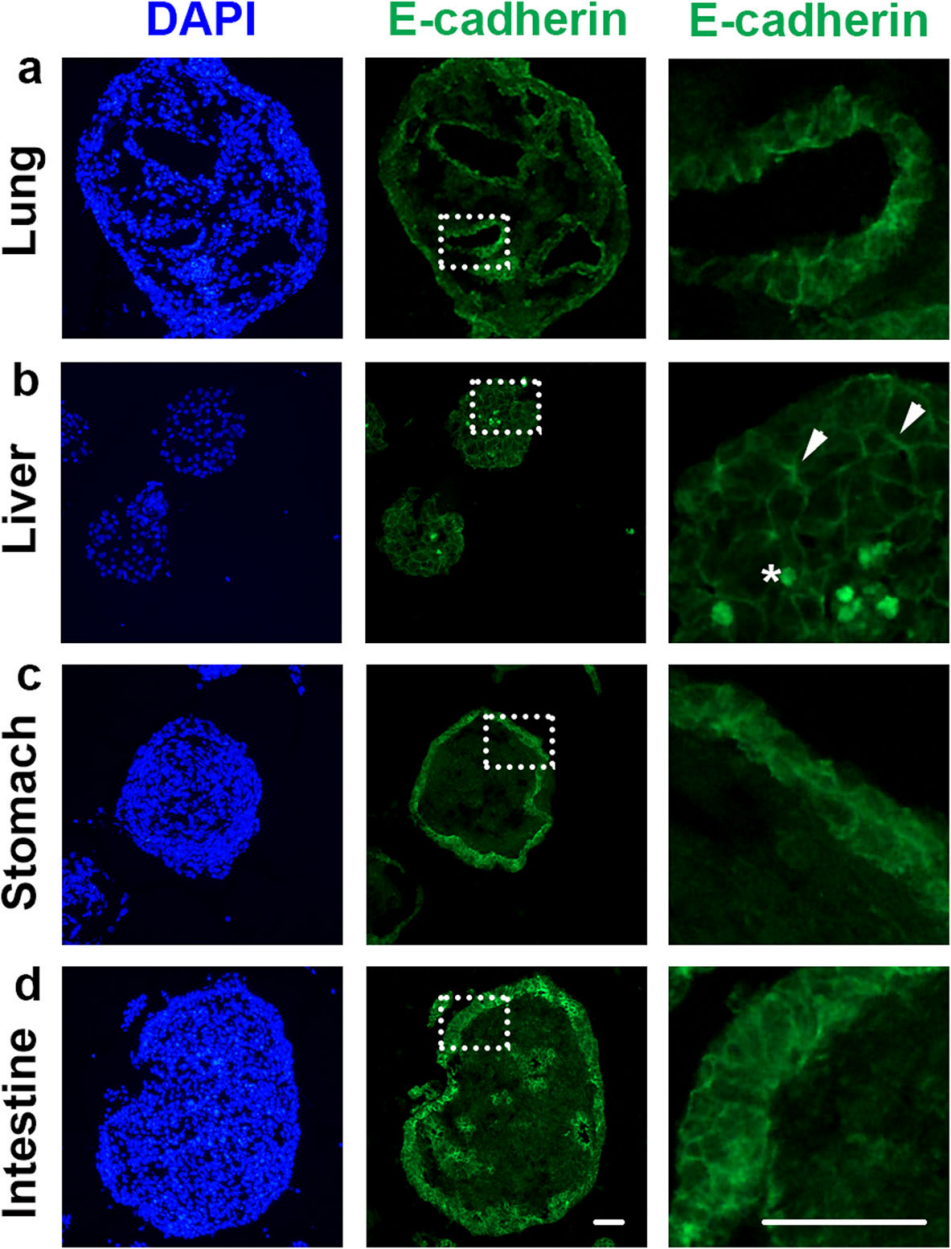
E-cadherin expression detected by immunocytochemistry in day 8 fetal lung, liver, stomach and intestinal spheroids. **a**, **c**, **d** In FOSs derived from lung, stomach and intestine (*n* = 7 for all), E-cadherin expression was mostly detected in the membranes of the outer cells of the spheroids. **b** In liver FOSs (*n* = 7), E-cadherin expression was detected in junctions between the cells throughout the spheroids (indicated by arrowheads). The asterisk indicates the presence of endogenous auto-fluorescence that is most likely due to lipofuscin accumulation. The right panels indicate a magnified view of the area highlighted by white dotted boxes in the middle panels. **a**-**d** DAPI indicates nuclei. Scale bars: 100 μm

Next, analyzes of typical tissue and/or cell markers by immunocytochemistry were performed on sectioned FOSs. Epithelial cadherin (encoded by *CDH1*), better known as E-cadherin, is the major adhesion component of epithelial adherens junctions that are essential for tissue barrier formation. E-cadherin plays an important role in epithelial tissues, such as lung, liver, stomach and intestine, for organ homeostasis during development and disease [34, 35]. Therefore, we examined E-cadherin expression in FOSs of lung, liver, stomach and intestine, and observed that all four FOSs expressed E-cadherin (Fig. 5). The liver FOSs showed E-cadherin expression throughout the whole spheroid structure (Fig. 5b), indicating membrane expression of hepatic epithelial cells, as well as clusters of auto-fluorescence (Fig. 5b), most likely caused by the accumulation of lipofuscin known to be present in Kupffer cells [36]. By contrast, lung, stomach and intestinal spheroids were lined by an epithelial-like E-cadherin^+^ layer, in a barrier-like formation (Fig. 5a, c, d).

**Fig. 6:**
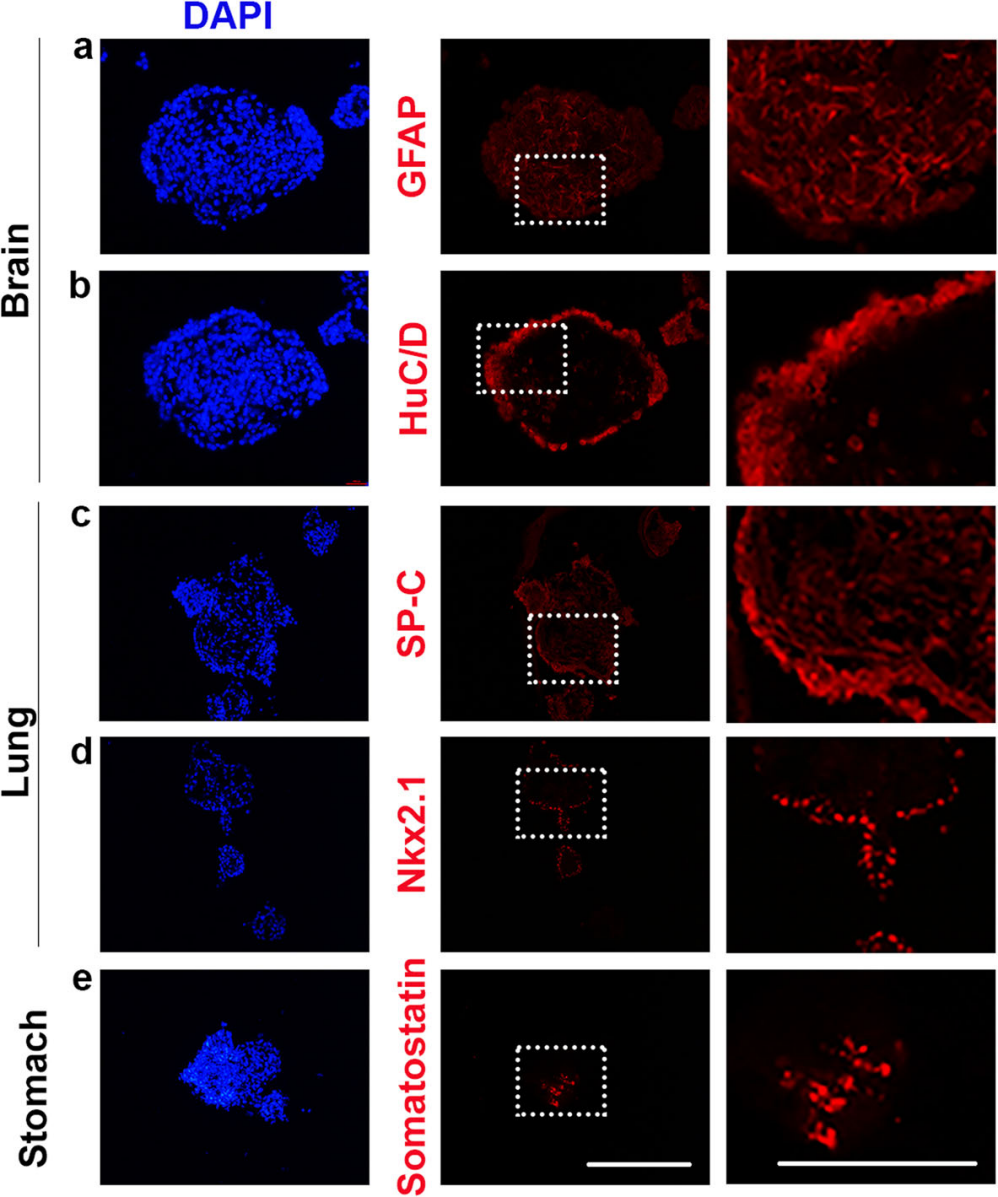
Typical cell marker expression detected by immunocytochemistry in day 8 fetal brain, lung and stomach spheroids. **a**, **b** Expression of GFAP, indicating astroglial cell types, and HuC/D, marking post-mitotic neurons, detected in FOSs derived from the brain (*n* = 9). **c**, **d** Expression of SP-C, defining Clara and/or alveolar type II cells, and Nkx2.1, indicating lung bud formation, observed in fetal lung spheroids (*n* = 6). **e** Expression of Somatostatin, defining δ-cells, detected in fetal stomach spheroids (*n* = 6). The right panels indicate a magnified view of the area highlighted by white dotted boxes in the middle panels. **a**-**d** DAPI indicates nuclei. Scale bars: 100 μm

To further exemplify how the FOSs can be characterized, we focused our analyzes to three FOSs; brain, lung and stomach. The presence of important differentiated cell types in these three spheroids was evaluated by immunocytochemistry. As expected, GFAP and the post-mitotic neuronal marker HuC/D, (encoded by *Elavl3/4*) [37], identifying astroglial cell types and post-mitotic neurons, respectively, were both expressed in brain FOSs (Fig. 6a, b). GFAP^+^ astroglial cell types were located throughout the entire spheroids, whereas the HuC/D^+^ neurons were located mostly in the periphery (Fig. 6a, b), indicating some extent of zonation in the 3D cellular organization of brain spheroids. In chick, the expression of *NK homeobox 2–1* (*Nkx2–1;* also called *thyroid transcription factor-1* [*TTF-1*]) is detected at the onset of lung bud formation and throughout lung development [38], and *Nkx2.1* knock-out mice are born dead due to lack of lung parenchyma [39]. Consistently, both SP-C and Nkx2.1 expression were detected in lung FOSs (Fig. 6c, d), with SP-C^+^ Clara and/or alveolar type II cells being observed broadly throughout the spheroids, and Nkx2.1 expression being restricted to the periphery (Fig. 6c, d). Gastrin, produced by G cells, is a hormone that regulates gastric acid production, which facilitates the digestion of proteins, as well as the absorption of various vitamins and minerals. To regulate gastric acid in the stomach, the peptide hormone Somatostatin, produced by δ-cells, acts to decrease acid production by preventing the expression and secretion of Gastrin. Somatostatin is also expressed in the gut of chick embryos [40]. Accordingly, Somatostatin+ δ-cells were observed in restricted clusters within stomach FOSs (Fig. 6e). Brain, lung and stomach FOSs exhibited the presence of tissue-specific differentiated cell-types that are consistent with their organ of origin. Collectively, this further validates the use of chick FOSs to mimic in vivo organs at the cellular level.

### Electron microscopy of FOSs

To study the morphological surface and cellular structures of different cell types present in the generated FOSs, EM was performed on brain and lung FOSs. Specifically, both scanning electron microscopy (SEM) and transmission electron microscopy (TEM; the Tokuyasu technique) were used to image fixed brain and lung FOSs.

**Fig. 7:**
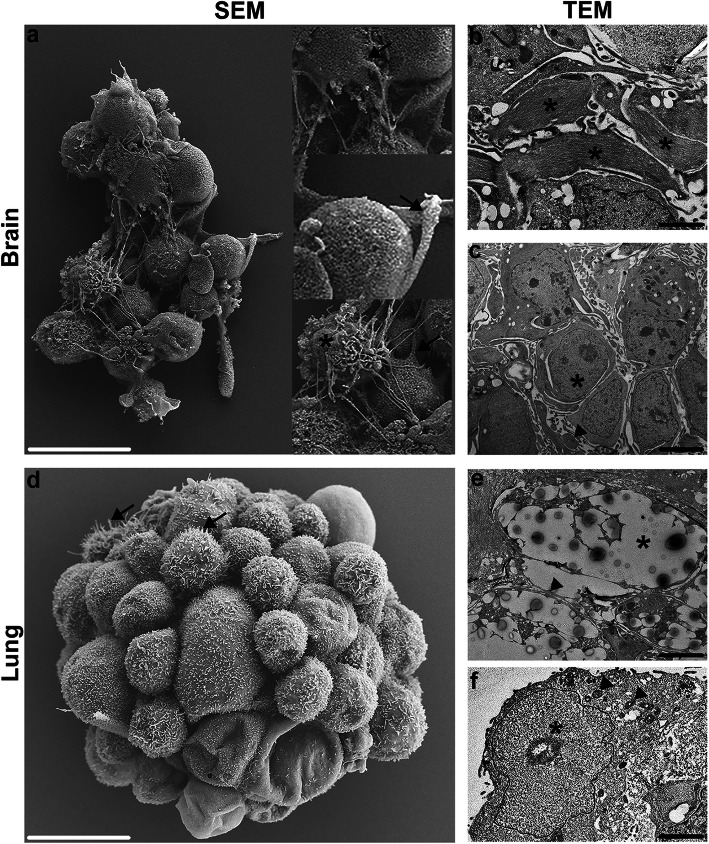
Electron microscopy (EM) of fetal brain and lung spheroids. **a**, **d** SEM and **b**, **c**, **e**, **f** TEM images of brain and lung spheroids. EM of fetal brain spheroids indicated the presence of nerve fibers (**a**, arrows in inserts), neuro-filaments (**b**, asterisks), astrocytes (**a**, **c**; asterisk) and dendrites (**c**, arrowhead). EM of fetal lung spheroids identified the presence of ciliated lung cells (**d**, arrows), alveolar lumen (**e**, asterisk) and alveolar septal walls (**e**, arrowheads), and lamellar bodies (**f**, arrow heads) in type II alveolar epithelial cells (**f**, asterisk). Scale bars: 10 μm (white), 2 μm (black)

During SEM processing, the large fetal brain spheroids (FBSs) were easily broken, whereas the smaller fetal lung spheroids (FLuSs) remained intact. SEM images of the fractioned FBSs showed nerve fibers between neuronal cells (Fig. 7a), indicating establishment of neuronal connections. Presumed astrocytes with characteristic multiple processes were also observed (Fig. 7a). Moreover, the TEM images of the FBSs indicated the presence of neuro-filament, astrocytes and dendrites (Fig. 7b, c). SEM views of FLuSs revealed cells with protrusions that are characteristic of ciliated lung cells (Fig. 7d). Furthermore, TEM images of the FLuSs indicated alveolar lumen, alveolar septal walls and lamellar bodies that are found in type II alveolar epithelial cells (Fig. 7e, f). As expected, the overall structural organization of spheroids derived from fetal brain and lung cells did not completely mimic in vivo organs, nevertheless, EM successfully identified important characteristic organ-specific structures in both brain and lung FOSs.

### Confrontation assay of FOSs with tumor spheres

To assess a functional application of FOSs, we performed a confrontation assay between specific FOSs and tumor spheres to analyze and monitor potential cell-cell contact and tumor invasion patterns. For this purpose, FBSs and FLuSs were confronted separately with tumor spheres derived from either the glioblastoma cell line U251 or the lung cancer cell line A549. To facilitate the monitoring of potential invasion, U251-*GFP* and A549-*GFP* cell lines that stably express green fluorescent protein (GFP) [17] were used to form spheres in a similar manner as described for the FOSs. Briefly, similarly sized FOSs and tumor spheres were placed together to establish a cellular confrontation, such that each well in an 8-well chamber contained one tumor sphere and one FOS. In total, four confrontation assays containing up to eight replicates each were seeded. After seeding, potential fusion between the tumor spheres and FOSs, and invasion of GFP-expressing cancer cells into or on the surface of the FOSs were examined by bright field and fluorescence microscopy, respectively, each day up to 10 days. In particular, image analyzes was focused at the interface between the tumor spheres and FOSs to determine the degree of cell-cell contact and tumor invasion.

**Fig. 8:**
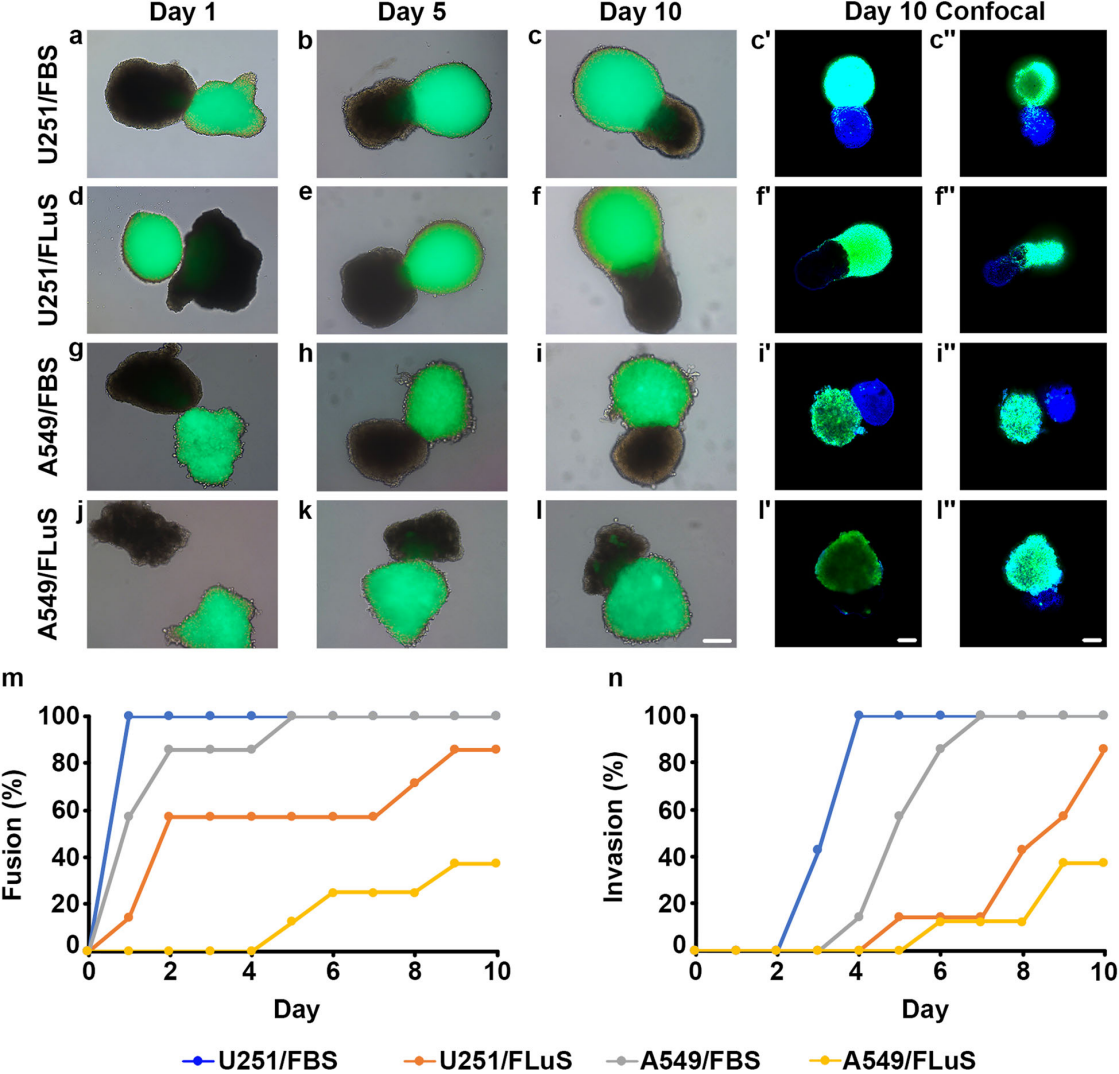
Confrontation assay. **a**-**l** Fetal brain and fetal lung spheroids (FBSs and FLuSs) were cultured separately in confrontation assays together with spheres of GFP-stably (green) expressing glioblastoma (U251) and lung cancer (A549) cells in different combinations. Fusion and invasion were monitored using bright field and fluorescence microscopy each day up to 10 days. **c**′, **c**′′, **f**′, **f**′′, **i**′ **i**′′, **l**′, **l**′′ At day 10, confocal imaging revealed tumor cell invasion into the FOSs, here shown at two different focal depths of the same fused sphere/spheroids pair. DAPI (blue) indicates nuclei. **m** Graph showing the percentage of fusion between cancer cell spheres and FOSs. **n** Graph showing the percentage of invasion of cancer cells into the FOSs. Scale bars: 200 μm (**a**-**l**′′)


Already at confrontation day 1, all U251 glioblastoma tumor spheres had fused with adjacent FBSs (Fig. 8a, m). Furthermore, fluorescence imaging provided evidence that from day 3, U251 cancer cells had started to invade the FBSs, a phenomenon observed in 100% of the confrontation pairs (*n* = 7; Fig. 8b-c′′, n and Movies S1-S2). Next, the combination of U251 tumor spheres and FLuS showed that 57% of the U251 spheres and FLuSs had fused at confrontation days 2–7, reaching 86% at day 10 (*n* = 7; Fig. 8d-f, m). Four out of seven U251 cancer spheres exhibited invasion patterns in combination with FLuS, starting from day 4 (Fig. 8f′, f′′, n and Movies S3-S4). Analyzing the A549 lung tumor sphere experiments showed that after 2–4 days, most (86%) A549 tumor spheres had fused with the FBSs, and thereafter a 100% of these fused tumor spheres: fetal spheroids was observed (*n* = 7; Fig. 8g-i, m). At day 7, tumor cells from all A549 spheres had invaded their paired FBSs (Fig. 8i′, i′′, n and Movies S5-S6). By contrast, up to 4 days, no fusion between A549 tumor spheres and FLuSs was observed, however, a minority (12.5–37.5%) of these tumor spheres did slowly fuse with fetal lung spheroids between day 5 and day 10 s (*n* = 8; Fig. 8j-l, m). Moreover, only minor invasion of A549 tumor cells into the FLuSs was detected during the 10 days of monitoring (Fig. 8l′, l′′, n and Movies S7-S8). Notably, the results indicated that U251 cells started to invade the FBSs within 1–2 days after fusion, whereas no or slower invasion (5–6 days) of U251 cells into the FLuSs was observed after fusion. Also, A549 cells fused to either FBSs or FLuS invaded the FOSs after 3–4 days (Fig. 8m and n). No evident morphological changes of the spheroids were observed during the duration of the confrontation assays.

**Additional file 2: Movie S1.** Video of confocal images of GFP-expressing U251 cancer cells invading the FBSs at day 10 of the confrontation assay (*n* = 7/7).


**Additional file 3: Movie S2.** Video of confocal images of GFP-expressing U251 cancer cells invading the FBSs at day 10 of the confrontation assay (*n* = 7/7). DAPI indicates cell nuclei.


**Additional file 4: Movie S3.** Video of confocal images of GFP-expressing U251 cancer cells invading the FLuSs at day 10 of the confrontation assay (*n* = 4/7).


**Additional file 5: Movie S4.** Video of confocal images of GFP-expressing U251 cancer cells invading the FLuSs at day 10 of the confrontation assay (*n* = 4/7). DAPI indicates cell nuclei.


**Additional file 6: Movie S5.** Video of confocal images of GFP-expressing A549 cancer cells invading the FBSs at day 10 of the confrontation assay (*n* = 7/7).


**Additional file 7: Movie S6.** Video of confocal images of GFP-expressing A549 cancer cells invading the FBSs at day 10 of the confrontation assay (*n* = 7/7). DAPI indicates cell nuclei.


**Additional file 8: Movie S7.** Video of confocal images of GFP-expressing A549 cancer cells invading the FLuSs at day 10 of the confrontation assay (*n* = 3/8).


**Additional file 9: Movie S8.** Video of confocal images of GFP-expressing A549 cancer cells invading the FLuSs at day 10 of the confrontation assay (*n* = 3/8). DAPI indicates cell nuclei.

Finally, confocal imaging indicated that the tumor cells invaded the organ spheroids, but not the other way around, as we did not detect any GFP-negative spheroid-derived cells in the tumor spheres (Movies S1-S8). Taken together these results indicate a cell-to-cell preference of tumor types and distinct target organs. This show that chick FOSs used in the confrontation assay may serve as a model to validate cell contact, cell fusion and tumor invasion, in which different molecular conditions that might alter fusion and/or invasive properties can be tested.

## Discussion

We have developed and validated an inexpensive, time efficient and easy methodology to maintain and utilize 3D organ spheroids in an anchorage-independent cell culture system, which allows for studies of specific organ characteristics in a dish. The established protocol enables time efficient, cost effective and long-term expansion of FOSs that could, at least in part, replace the use of the more expensive and complex organoid models to study critical processes in development and disease.

Pierzchalska and co-workers have published several studies regarding protocols for the generation of chick intestinal organoids [41–43], and other specific methods for generating individual 3D organoids using chick, mouse or rat embryonic tissue have also been reported (reviewed in [44]). In contrast, our FOS model is the first general method to systematically generate 3D cell-based organ spheroids from multiple chick embryonic organs. Moreover, the FOS model is not restricted to any specific stage or organ. One major advantage using cells from chick embryos younger than E14, compared to rodent or human cells, is that these experiments do not require ethical permission. Challenges with our newly established FOS assay and previous spheroid models are foremost an uncontrolled cellular organization within the spheroids themselves. As for many 3D in vitro organ models, FOSs also lack the in vivo context of adjacent tissues, which might be a disadvantage, but the removal of potential confounding secondary effects might also offer advantages over more multifaceted and interdependent organoid systems. As such, chick FOSs could be considered a suitable first model system before performing more expensive and complex in vivo experiments.

Our aim of establishing a broad ranging and highly effective method to generate FOSs from different organs, here including brain, lung, heart, liver, stomach, intestine, and epidermis, was successful in terms of the formation of healthy, proliferating 3D organ spheroids that exhibited organ-like characteristics at both molecular and cellular levels. The combined results from studying cell growth, renewal and morphology, in part using SEM and TEM, as well as gene and protein expression via RT-PCR and immunohistochemistry, indicates that the variety of generated FOSs in this study exhibit organ characteristics that mimicked those of their corresponding in vivo organs. Future studies will include more in-depth analyzes of the individual FOSs from an organ perspective when viewed with a particular developmental or disease approach in mind. Such work has previously been presented for kidney organoids that were established from embryonic mouse metanephric mesenchyme and Renca cells to study kidney cancer [45]; cerebral cortex, brainstem and spinal cord aggregates that were developed from relevant mouse fetal neural tissue to study organotypic synaptic networks [46]; intestinal spheroids/organoids that were established from isolated mouse crypt cells to study maturation of the intestine [47, 48]; and pancreatic spheres/organoids that were generated from mouse embryonic pancreas to study normal pancreas development [49].

Both 2D and 3D cell cultures are important models for studies including stem cells, development, cancer and other types of disease research, toxicology as well as drug discovery. However, 2D cultures have many limitations that are related to their failure to mimic in vivo conditions for polarity, cellular organization and extracellular environments. In the 1970s, the first 3D cultures were established using human tumor stem cells in soft agar solution [50], with various 3D cell culture systems being developed during the last couple of decades to overcome these shortcomings [51, 52]. Today, a well-known 3D model is the organoid, which is defined as a collection of organ-specific cell types that develops from stem cells or organ progenitors and self-organizes through cell sorting and spatially restricted lineage commitment in a manner similar to the in vivo situation [53]. Most, if not all, organoid cultures require an anchoring scaffold, such as fibrinogen, collagen, Matrigel or synthetic hydrogels [52]. By contrast, our newly established FOS approach is independent on an external matrix for growth, and the cells are therefore not exposed to or artificially affected by signals that might derive from the anchoring scaffold itself. This is accomplished by culturing fetal cells on ultra-low attachment coating, in our case 1.5% agarose, which prevents cell attachment to the plastic surface and, thereby, forces cells to aggregate in the suspension. Other scaffold-free 3D cell culture techniques are the hanging drop method, magnetic levitation and microfluidic systems [52]. The hanging drop method is based on spheroid formation through the impact of gravity in drops of cell suspension on a Petri dish lid turned upside-down, which also avoids the need for a plate coating. Similar to our newly established FOS technique, an advantage with the hanging drop method, is the fast formation of spheroids. However, a clear limitation with the hanging drop approach is the difficulty to exchange cell culture media in the drops, which reduces culture time to only 1–2 weeks [54]. Our method, on the other hand, permits the maintenance of some FOSs for at least a month. Magnetic levitation involves loading cells with magnetic nanoparticles, which subsequently are cultured in ultra-low attachment plates under the exposure to an external magnetic field that cause cell aggregation and spheroid formation [55]. A few disadvantages with this method, apart from the absolute requirement for expensive magnetic beads that at high concentrations can be toxic to cells, include overriding the endogenous aggregate mechanisms of cells and the potential risk of perturbing normal inter- and intracellular molecular mechanisms. Microfluidic systems or “organ-on-a chip” platforms [56, 57], utilize cells that are cultured in micro-chambers in media that constantly infuses the chambers. The advantage here is the intrinsic use of fluid flow instead of still media, but a drawback is the technically more complex culture system including difficulties collecting spheroids for analyses. By comparison, the FOS approach is an excellent complement in the arsenal of 3D cell culture systems in terms of feasibility (low cost, simplicity), viability (high yield of spheroid formation, robustness of spheroids) and longevity (the possibility to culture spheroids for several weeks up to months).

Developmental studies is a key area for which chick FOSs could serve as a model. It is technically challenging to electroporate chick embryos in ovo beyond E3, and ex ovo beyond E7, to target distinct internal tissues and organs without damaging the embryo or targeting other structures [58]. As such, FOSs could act as an alternative developmental assay to modulate gene expression in distinct organs to follow the development of organ specific cell types. Moreover, the intrinsic development of cell types in FOSs can be followed without the influence of secondary effects from nearby tissues.

A critical functional application of the FOS method presented here might be within cancer research, where our results show clearly that cell-to-cell contact, fusion and invasion can be studied using a confrontation assay between a multitude of organ spheroid and tumor sphere combinations. This is particularly relevant to metastasis, which typically involves multiple steps, including invasion [59], and is the main cause of cancer-related mortality. Thus, the presented confrontation assay may be useful as an initial functional method before proceeding to more complex, time-consuming and expensive in vivo cancer experiments. Accumulating evidence indicates that tumor system support the “seed and soil” hypothesis on the non-random distributions of metastasis [60], in which unique properties of particular tumor cells (seed) and different characteristics of each organ microenvironment (soil) collectively determine the organ preference of metastasis [61, 62]. Our observation that U251 glioblastoma cells infiltrate the brain spheroids faster and more extensively compared to the lung spheroids is in concordance with the fact that glioblastoma is highly invasive locally within the brain, but extracranial metastasis is a rare event [17, 63]. On the other hand, the A549 lung cancer cells fused and infiltrated the brain spheroids earlier than the lung spheroids, which likely reflects the higher metastatic capacity of this type of cancer cells [17, 64]. Together our results indicate a cell-to-cell preference between tumor types and distinct target organs, which supports the “seed and soil” hypothesis, and show that our chick organ spheroid/tumor sphere confrontation assay can be used to investigate the interaction of tumor cells with specific tissue or cell types, as well as the mechanisms that govern tumor invasion.

Potentially, a FOS-based system might also be an appropriate model to analyze cell contact and viral infection patterns, for example, to investigate whether a virus infects cells uniformly, in a cell specific manner and/or how the infection progresses. Indeed, chick embryos have been used previously to examine molecular regulations in distinct virus infections, for example the Zika virus [65], the herpes simplex virus [66], and the influenza A virus [67]. Given the current worldwide Covid19 pandemic caused by the SARS-CoV-2 coronavirus and the escalation of multisystem involvement, as well as the increased risk of future novel outbreaks, the application of low-cost, reliable and reproducible FOS-based assays that utilize different organ systems in determining key virus:host cell interactions might offer significant breakthroughs in the generation of new knowledge in this area.

## Conclusions

In general, 3D models are of great value in research related to development, disease, drug testing and tissue engineering as they reflect physiological conditions more efficiently than 2D cell cultures, thus providing a more predictive data for future in vivo tests. Our data illustrates that the newly established chick FOS assay can be used for developmental studies, as well as to evaluate both cell-to-cell interactions and invasion properties of cancer cells. With further analyzes, we consider that our chick FOS method will be applicable in many other scientific areas such as viral infections, drug screening, cancer diagnostics and/or tissue engineering.

## Supplementary Information

**Additional file 1: Fig. S1.** Schematic overview illustrating the generation of chick FOS. (a) Five samples of brain, heart, liver, stomach and epidermis were collected from E12, and (b) five samples of lung and intestine E14 chick embryos. (c) FOSs workflow (steps 1–4), from harvesting organs to seeding cells in culture flasks. (d) A table indicating the duration of Accutase incubation required to dissociate specific organs into a cell suspension depending on the tissue structure (step 3 in c). Scale bars: 5 mm (white), 2 mm (black). **Fig. S2.** Day 1 of FOSs cultures. (a-g) Cells from all fetal organs; brain, lung, heart, liver, stomach, intestine and epidermis, formed spheroid-like structures after 1 day in culture. Scale bar: 100 μm. **Fig. S3.** Sox2 expression analyzed by immunocytochemistry in day 8 in FOSs. (a-c) No expression of Sox2 was detected in day 8 fetal heart, intestinal or epidermal spheroids (*n* = 7 for all). DAPI indicates nuclei. Scale bar: 100 μm. **Fig. S4.** Expression of cleaved (c) Caspase3 by immunocytochemistry in day 8 FOSs. (a-g) Only a few cCasp3^+^ cells, indicative of apoptotic cells, were observed in FOSs derived from the seven analyzed organs (*n* = 5 for all FOSs). DAPI indicates nuclei. Scale bar: 100 μm. **Fig. S5.** Full-length gel images of gene expression in seven different FOSs and their corresponding chick embryo in vivo organs. (a-i) RT-PCR of day 8 FOSs revealed that mRNA expression of typical organ characteristic genes are enriched in the different FOSs. (a) *GFAP* expression in brain, but not in heart or liver FOSs/organs. (b) *SP-C* expression in lung, but not in heart or stomach FOSs/organs. (c) *TNNT2* expression in heart, but not in brain or stomach FOSs/organs. (d) *Alb* expression in liver, but not in lung or heart FOSs/organs. (e) *Barx1* expression in stomach, but not in brain or lung FOSs/organs. (f) *SI* expression in intestine, but not in brain or heart FOSs/organs. (g) *EDMTFH* expression in epidermis, but not in lung or liver FOSs/organs. (h, i) The house-keeping gene *GAPDH* was used as a reference gene. (a-i) The first and last lane of each gel image show the ladder, and an amplicon size of 1500 base pairs (bp) are indicated. **Fig. S6.** Full-length gel images of RT-PCR *Vimentin* expression in seven different FOSs and their corresponding chick embryo in vivo organs. (a, b) RT-PCR revealed mRNA expression of *Vimentin* (*Vim*) in all day 8 FOSs (a), and in their corresponding embryonic in vivo organs (b); E12 brain, heart, liver, stomach and epidermis, and E14 lung and intestine. The house-keeping gene *GAPDH* was used as a reference. The first lane of each gel image shows the ladder, and an amplicon size of 1500 base pairs (bp) is indicated. **Table S1.** Perimeter of fetal organ spheroids at culture day 4, 8 and 12. All measurements are expressed in mm and stated as mean ± standard error of the mean (SEM). Δp = fold change of the mean perimeter between FOSs measured on the stated days. **Table S2.** Genes detected by RT-PCR. Sequences for each primer pair (forward, F, reverse, R), with indicated amplicon sizes (base pairs, bp) and annealing temperatures (T; °C).

## Data Availability

Raw data were generated at Umeå University, Sweden, where all cell lines and FOSs sections are properly stored. When relevant, derived data supporting the findings of this study are available from the corresponding author (LG) upon request.

## Abbreviations

2D: Two-dimensional
3D: Three-dimensional
cDNA: Complementary DNA
Alb: Albumin
ATCC: American Type Culture Collection
Barx1: Homeobox protein BarH-like 1
D: Day
DAPI: 4′:6-diamidino-2-phenylindole, dihydrochloride
DSHB: Developmental Studies Hybridoma Bank
E: Embryonic day
ECACC: European Collection of Authenticated Cell Cultures
EDMTFH: Epidermal differentiation protein starting with a MTF motif and rich in histidine
EM: Electron microscopy
ESCs: Embryonic stem cells
FBS: Fetal bovine serum
FBS: Fetal brain spheroids
FESEM: Field-emission scanning electron microscopy
FLuS: Fetal lung spheroids
FOSs: Fetal organ spheroids
GA: Glutaraldehyde
GAPDH: Glyceraldehyde-3-phosphate dehydrogenase
GFAP: Glial fibrillary acidic protein
GFP: Green fluorescent protein
h: Hour/s
HuC/D (also Elavl3/4): Neural Hu C/D protein
ICC: Immunocytochemistry
iPSCs: Induced pluripotent stem cells
min: Minute/s
n: Number
Nkx2.1 (also TTF-1): NK2 homeobox 1 (also thyroid transcription factor-1)
ON: Overnight
PB: Phosphate buffer
PBS: Phosphate-buffered saline
PFA: Paraformaldehyde
pHH3: Phosphorylated histone H3
PS: Penicillin-streptomycin
RT: Room temperature
RT-PCR: Reverse transcription –PCR
s: Second/s
SEM: Scanning electron microscopy
SI: Sucrose-isomaltase
Sox2: SRY (sex determining region Y) box 2
SP-C (also SFTPC): Surfactant protein C
T°C: Annealing temperature
TEM: Transmission electron microscopy
TNNT2 (also cTnT): Cardiac muscle troponin T2
Vim: Vimentin

## References

[1] Rashidi H, Sottile V (2009). The chick embryo: hatching a model for contemporary biomedical research. Bioessays.

[2] Stern CD (2005). The chick; a great model system becomes even greater. Dev Cell.

[3] Dodgson JB, Romanov MN (2004). Use of chicken models for the analysis of human disease. Curr Protoc Hum Genet.

[4] Drillon G, Champeimont R, Oteri F, Fischer G, Carbone A (2020). Phylogenetic reconstruction based on Synteny block and gene adjacencies. Mol Biol Evol.

[5] Green RE, Braun EL, Armstrong J, Earl D, Nguyen N, Hickey G, Vandewege MW, St John JA, Capella-Gutierrez S, Castoe TA (2014). Three crocodilian genomes reveal ancestral patterns of evolution among archosaurs. Science.

[6] Meadows JRS, Lindblad-Toh K (2017). Dissecting evolution and disease using comparative vertebrate genomics. Nat Rev Genet.

[7] Eiraku M, Takata N, Ishibashi H, Kawada M, Sakakura E, Okuda S, Sekiguchi K, Adachi T, Sasai Y (2011). Self-organizing optic-cup morphogenesis in three-dimensional culture. Nature.

[8] Lancaster MA, Renner M, Martin CA, Wenzel D, Bicknell LS, Hurles ME, Homfray T, Penninger JM, Jackson AP, Knoblich JA (2013). Cerebral organoids model human brain development and microcephaly. Nature.

[9] Huch M, Knoblich JA, Lutolf MP, Martinez-Arias A (2017). The hope and the hype of organoid research. Development.

[10] Huch M, Koo BK (2015). Modeling mouse and human development using organoid cultures. Development.

[11] Lancaster MA, Knoblich JA (2014). Generation of cerebral organoids from human pluripotent stem cells. Nat Protoc.

[12] Kim J, Koo BK, Knoblich JA (2020). Human organoids: model systems for human biology and medicine. Nat Rev Mol Cell Biol.

[13] Lancaster MA, Huch M. Disease modelling in human organoids. Dis Model Mech. 2019;12(7):dmm039347. https://doi.org/10.1242/dmm.039347. 10.1242/dmm.039347 PMC6679380 31383635

[14] Hamburger V, Hamilton HL (1951). A series of normal stages in the development of the chick embryo. J Morphol.

[15] Wittmann W, Iulianella A, Gunhaga L (2014). Cux2 acts as a critical regulator for neurogenesis in the olfactory epithelium of vertebrates. Dev Biol.

[16] Tokuyasu KT (1973). A technique for ultracryotomy of cell suspensions and tissues. J Cell Biol.

[17] Palaniappan TK, Slekiene L, Jonasson AK, Gilthorpe J, Gunhaga L (2020). CAM-Delam: an in vivo approach to visualize and quantify the delamination and invasion capacity of human cancer cells. Sci Rep.

[18] Schneider CA, Rasband WS, Eliceiri KW (2012). NIH image to ImageJ: 25 years of image analysis. Nat Methods.

[19] Sholl-Franco A, Fragel-Madeira L, Macama Ada C, Linden R, Ventura AL (2010). ATP controls cell cycle and induces proliferation in the mouse developing retina. Int J Dev Neurosci.

[20] Jean C, Oliveira NM, Intarapat S, Fuet A, Mazoyer C, De Almeida I, Trevers K, Boast S, Aubel P, Bertocchini F (2015). Transcriptome analysis of chicken ES, blastodermal and germ cells reveals that chick ES cells are equivalent to mouse ES cells rather than EpiSC. Stem Cell Res.

[21] Sarkar A, Hochedlinger K (2013). The sox family of transcription factors: versatile regulators of stem and progenitor cell fate. Cell Stem Cell.

[22] Kalman M, Szekely AD, Csillag A (1993). Distribution of glial fibrillary acidic protein-immunopositive structures in the brain of the domestic chicken (Gallus domesticus). J Comp Neurol.

[23] Norkute A, Kipp M, Dang J, von Keyserlingk DG, Valanciute A, Beyer C (2010). Early formation of a GFAP-positive cell population in the ventricular zone during chicken brain development. Cells Tissues Organs.

[24] Mailleux AA, Tefft D, Ndiaye D, Itoh N, Thiery JP, Warburton D, Bellusci S (2001). Evidence that SPROUTY2 functions as an inhibitor of mouse embryonic lung growth and morphogenesis. Mech Dev.

[25] Boggaram V (2003). Regulation of lung surfactant protein gene expression. Front Biosci.

[26] England J, Pang KL, Parnall M, Haig MI, Loughna S (2016). Cardiac troponin T is necessary for normal development in the embryonic chick heart. J Anat.

[27] Dimattia GE, Lazier CB (1993). Expression of the albumin gene in the yolk sac and liver during chick embryogenesis. Comp Biochem Physiol B.

[28] Smith DM, Grasty RC, Theodosiou NA, Tabin CJ, Nascone-Yoder NM (2000). Evolutionary relationships between the amphibian, avian, and mammalian stomachs. Evol Dev.

[29] Kim BM, Buchner G, Miletich I, Sharpe PT, Shivdasani RA (2005). The stomach mesenchymal transcription factor Barx1 specifies gastric epithelial identity through inhibition of transient Wnt signaling. Dev Cell.

[30] Karasov WH, Caviedes-Vidal E (2021). Adaptation of intestinal epithelial hydrolysis and absorption of dietary carbohydrate and protein in mammals and birds. Comp Biochem Physiol A Mol Integr Physiol.

[31] Alibardi L, Holthaus KB, Sukseree S, Hermann M, Tschachler E, Eckhart L (2016). Immunolocalization of a histidine-rich epidermal differentiation protein in the chicken supports the hypothesis of an evolutionary developmental link between the embryonic subperiderm and feather barbs and barbules. PLoS One.

[32] Loe AKH, Rao-Bhatia A, Kim JE, Kim TH (2021). Mesenchymal niches for digestive organ development, homeostasis, and disease. Trends Cell Biol.

[33] Page M (1989). Changing patterns of cytokeratins and vimentin in the early chick embryo. Development.

[34] Bruner HC, Derksen PWB. Loss of E-Cadherin-Dependent Cell-Cell Adhesion and the Development and Progression of Cancer. Cold Spring Harb Perspect Biol. 2018;10(3):a029330. 10.1101/cshperspect.a029330. 10.1101/cshperspect.a029330 PMC5830899 28507022

[35] Daulagala AC, Bridges MC, Kourtidis A. E-cadherin Beyond Structure: A Signaling Hub in Colon Homeostasis and Disease. Int J Mol Sci. 2019;20(11):2756. 10.3390/ijms20112756. 10.3390/ijms20112756 PMC6600153 31195621

[36] Kong XY, Nesset CK, Damme M, Loberg EM, Lubke T, Maehlen J, Andersson KB, Lorenzo PI, Roos N, Thoresen GH (2014). Loss of lysosomal membrane protein NCU-G1 in mice results in spontaneous liver fibrosis with accumulation of lipofuscin and iron in Kupffer cells. Dis Model Mech.

[37] Alpar A, Kunzle H, Gartner U, Popkova Y, Bauer U, Grosche J, Reichenbach A, Hartig W (2010). Slow age-dependent decline of doublecortin expression and BrdU labeling in the forebrain from lesser hedgehog tenrecs. Brain Res.

[38] Zeng X, Yutzey KE, Whitsett JA (1998). Thyroid transcription factor-1, hepatocyte nuclear factor-3beta and surfactant protein a and B in the developing chick lung. J Anat.

[39] Kimura S, Hara Y, Pineau T, Fernandez-Salguero P, Fox CH, Ward JM, Gonzalez FJ (1996). The T/ebp null mouse: thyroid-specific enhancer-binding protein is essential for the organogenesis of the thyroid, lung, ventral forebrain, and pituitary. Genes Dev.

[40] Rawdon BB, Andrew A (1999). Gut endocrine cells in birds: an overview, with particular reference to the chemistry of gut peptides and the distribution, ontogeny, embryonic origin and differentiation of the endocrine cells. Prog Histochem Cytochem.

[41] Panek M, Grabacka M, Pierzchalska M (2018). The formation of intestinal organoids in a hanging drop culture. Cytotechnology.

[42] Pierzchalska M, Grabacka M, Michalik M, Zyla K, Pierzchalski P (2012). Prostaglandin E2 supports growth of chicken embryo intestinal organoids in Matrigel matrix. Biotechniques.

[43] Pierzchalska M, Panek M, Grabacka M (2019). The migration and fusion events related to ROCK activity strongly influence the morphology of chicken embryo intestinal organoids. Protoplasma.

[44] Lou YR, Leung AW (2018). Next generation organoids for biomedical research and applications. Biotechnol Adv.

[45] Xu Q, Junttila S, Scherer A, Giri KR, Kivela O, Skovorodkin I, Roning J, Quaggin SE, Marti HP, Shan J (2017). Renal carcinoma/kidney progenitor cell chimera organoid as a novel tumorigenesis gene discovery model. Dis Model Mech.

[46] Crain SM, Bornstein MB (1972). Organotypic bioelectric activity in cultured reaggregates of dissociated rodent brain cells. Science.

[47] Mustata RC, Vasile G, Fernandez-Vallone V, Strollo S, Lefort A, Libert F, Monteyne D, Perez-Morga D, Vassart G, Garcia MI (2013). Identification of Lgr5-independent spheroid-generating progenitors of the mouse fetal intestinal epithelium. Cell Rep.

[48] Navis M, Martins Garcia T, Renes IB, Vermeulen JL, Meisner S, Wildenberg ME, et al. Mouse fetal intestinal organoids: new model to study epithelial maturation from suckling to weaning. EMBO Rep. 2019;20(2):e46221. 10.15252/embr.201846221. 10.15252/embr.201846221 PMC6362357 30530633

[49] Greggio C, De Franceschi F, Figueiredo-Larsen M, Grapin-Botton A. In vitro pancreas organogenesis from dispersed mouse embryonic progenitors. J Vis Exp. 2014;89:51725. 10.3791/51725. 10.3791/51725 PMC4220853 25079453

[50] Hamburger AW, Salmon SE (1977). Primary bioassay of human tumor stem cells. Science.

[51] Foglietta F, Canaparo R, Muccioli G, Terreno E, Serpe L (2020). Methodological aspects and pharmacological applications of three-dimensional cancer cell cultures and organoids. Life Sci.

[52] Jensen C, Teng Y (2020). Is it time to start transitioning from 2D to 3D cell culture?. Front Mol Biosci.

[53] Lancaster MA, Knoblich JA (2014). Organogenesis in a dish: modeling development and disease using organoid technologies. Science.

[54] Lee WG, Ortmann D, Hancock MJ, Bae H, Khademhosseini A (2010). A hollow sphere soft lithography approach for long-term hanging drop methods. Tissue Eng Part C Methods.

[55] Souza GR, Molina JR, Raphael RM, Ozawa MG, Stark DJ, Levin CS, Bronk LF, Ananta JS, Mandelin J, Georgescu MM, Bankson JA, Gelovani JG, Killian TC, Arap W, Pasqualini R (2010). Three-dimensional tissue culture based on magnetic cell levitation. Nat Nanotechnol.

[56] Bhatia SN, Ingber DE (2014). Microfluidic organs-on-chips. Nat Biotechnol.

[57] Kwapiszewska K, Michalczuk A, Rybka M, Kwapiszewski R, Brzozka Z (2014). A microfluidic-based platform for tumour spheroid culture, monitoring and drug screening. Lab Chip.

[58] Luo J, Yan X, Lin J, Rolfs A. Gene transfer into older chicken embryos by ex ovo electroporation. J Vis Exp. 2012;65:4078. 10.3791/4078. 10.3791/4078 PMC3476417 22872055

[59] Massague J, Obenauf AC (2016). Metastatic colonization by circulating tumour cells. Nature.

[60] Akhtar M, Haider A, Rashid S, Al-Nabet A (2019). Paget's "seed and soil" theory of Cancer metastasis: an idea whose time has come. Adv Anat Pathol.

[61] Langley RR, Fidler IJ (2011). The seed and soil hypothesis revisited--the role of tumor-stroma interactions in metastasis to different organs. Int J Cancer.

[62] Nicolson GL (1988). Organ specificity of tumor metastasis: role of preferential adhesion, invasion and growth of malignant cells at specific secondary sites. Cancer Metastasis Rev.

[63] Liu B, Wang L, Shen LL, Shen MZ, Guo XD, Wang T, Liang QC, Wang C, Zheng J, Li Y, Jia LT, Zhang H, Gao GD (2012). RNAi-mediated inhibition of presenilin 2 inhibits glioma cell growth and invasion and is involved in the regulation of Nrg1/ErbB signaling. Neuro-Oncology.

[64] Qin T, Huang D, Liu Z, Zhang X, Jia Y, Xian CJ, Li K (2018). Tumor necrosis factor superfamily 15 promotes lymphatic metastasis via upregulation of vascular endothelial growth factor-C in a mouse model of lung cancer. Cancer Sci.

[65] Thawani A, Sirohi D, Kuhn RJ, Fekete DM (2018). Zika virus can strongly infect and disrupt secondary organizers in the ventricular zone of the embryonic chicken brain. Cell Rep.

[66] Hafezi W, Lorentzen EU, Eing BR, Muller M, King NJ, Klupp B, Mettenleiter TC, Kuhn JE (2012). Entry of herpes simplex virus type 1 (HSV-1) into the distal axons of trigeminal neurons favors the onset of nonproductive, silent infection. PLoS Pathog.

[67] Hussain S, Turnbull ML, Wise HM, Jagger BW, Beard PM, Kovacikova K, et al. Mutation of influenza A virus PA-X decreases pathogenicity in chicken embryos and can increase the yield of reassortant candidate vaccine viruses. J Virol. 2019;93(2):e01551–18. 10.1128/JVI.01551-18. 10.1128/JVI.01551-18 PMC6321911 30381488

