# Supplementary material for: Chick fetal organ spheroids as a model to study development and disease
Source: BMC Mol Cell Biol. 2021 Jul 5;22:37. doi: 10.1186/s12860-021-00374-6 (PMC8256237; doi:10.1186/s12860-021-00374-6)
Supplement: Supplementary file 9 — Additional file 1: Fig. S1. Schematic overview illustrating the generation of chick FOS. (a) Five samples of brain, heart, liver, stomach and epidermis were collected from E12, and (b) five samples of lung and intestine E14 chick embryos. (c) FOSs workflow (steps 1–4), from harvesting organs to seeding cells in culture flasks. (d) A table indicating the duration of Accutase incubation required to dissociate specific organs into a cell suspension depending on the tissue structure (step 3 in c). Scale bars: 5 mm (white), 2 mm (black). Fig. S2. Day 1 of FOSs cultures. (a-g) Cells from all fetal organs; brain, lung, heart, liver, stomach, intestine and epidermis, formed spheroid-like structures after 1 day in culture. Scale bar: 100 μm. Fig. S3. Sox2 expression analyzed by immunocytochemistry in day 8 in FOSs. (a-c) No expression of Sox2 was detected in day 8 fetal heart, intestinal or epidermal spheroids (n = 7 for all). DAPI indicates nuclei. Scale bar: 100 μm. Fig. S4. Expression of cleaved (c) Caspase3 by immunocytochemistry in day 8 FOSs. (a-g) Only a few cCasp3+ cells, indicative of apoptotic cells, were observed in FOSs derived from the seven analyzed organs (n = 5 for all FOSs). DAPI indicates nuclei. Scale bar: 100 μm. Fig. S5. Full-length gel images of gene expression in seven different FOSs and their corresponding chick embryo in vivo organs. (a-i) RT-PCR of day 8 FOSs revealed that mRNA expression of typical organ characteristic genes are enriched in the different FOSs. (a) GFAP expression in brain, but not in heart or liver FOSs/organs. (b) SP-C expression in lung, but not in heart or stomach FOSs/organs. (c) TNNT2 expression in heart, but not in brain or stomach FOSs/organs. (d) Alb expression in liver, but not in lung or heart FOSs/organs. (e) Barx1 expression in stomach, but not in brain or lung FOSs/organs. (f) SI expression in intestine, but not in brain or heart FOSs/organs. (g) EDMTFH expression in epidermis, but not in lung or liver FOSs/organs. (h, [file 12860_2021_374_MOESM1_ESM.pdf]

Supplementary Material for

Chick fetal organ spheroids as a model to study development and disease

Including:

Abbreviations

Figures S1-S6

Table S1-S2

Movie Captions S1-S8

by

Soran Dakhel[1,2], Wayne I. L. Davies[1], Justin V. Joseph[1], Tushar Tomar[3], Silvia Remeseiro[1,2], and Lena Gunhaga*[1]

[1]Umeå Centre for Molecular Medicine, Umeå University, 901 87, Umeå, Sweden

[2]Wallenberg Centre for Molecular Medicine, Umeå University, 901 87, Umeå, Sweden

[3]PamGene International B.V., Wolvenhoek 10, 5211 HH´s-Hertogenbosch, The Netherlands

*e-mail: lena.gunhaga@umu.se

Abbreviations

Δp = fold difference in the mean perimeter; *Alb: albumin;* B: brain; bp: base pairs; Barx1: *Homeobox protein BarH-like 1*; cCaspase3: cleaved caspase3; D: day; DAPI: 4':6-diamidino-2-phenylindole, dihydrochloride; E: embryonic day; E: epidermis; EDMTFH: epidermal differentiation protein starting with a MTF motif and rich in histidine; F: forward primer; FBS: fetal brain spheroids; FLuS: fetal lung spheroids; FOSs: fetal organ spheroids; GAPDH: glyceraldehyde-3-phosphate dehydrogenase; GFAP: glial fibrillary acidic protein; GFP: green fluorescent protein; H: heart; I: intestine; Lg: lung; Lv: liver; min: minute/s; n: number; PCR: polymerase chain reaction; R: reverse primer; RT-PCR: reverse transcription PCR; S: stomach; SEM: standard error of the mean; SI: sucrose-isomaltase; Sox2: SRY (sex determining region Y) box 2; SP-C (also SFTPC): surfactant protein C; T°C: annealing temperature; *TNNT2 (also cTnT): cardiac muscle troponin T2*; Vim: vimentin.

Figure S1

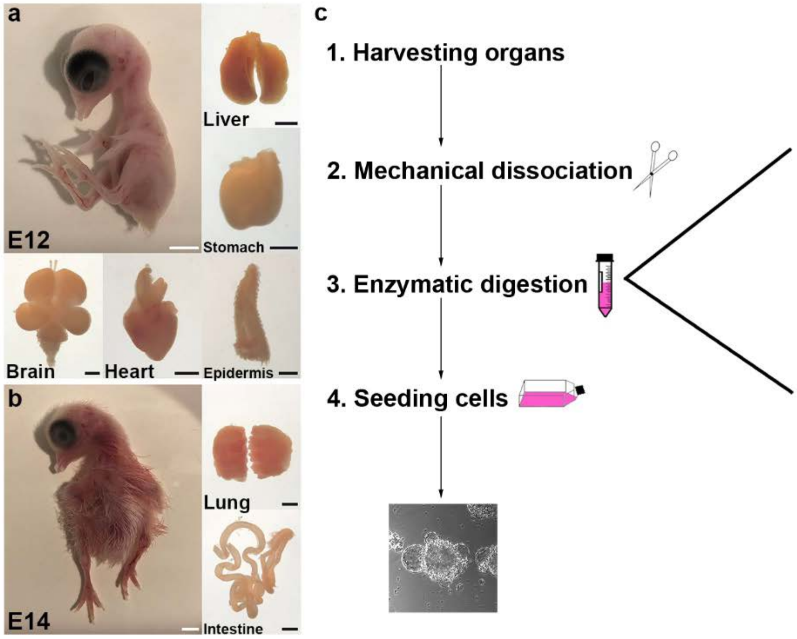

d

Organ	Accutase inc. time (min)
Brain	5
Lung	10
Heart	10
Liver	10
Stomach	15
Intestine	20
Epidermis	15

Fig. S1 Schematic overview illustrating the generation of chick FOS. (a) Five samples of brain, heart, liver, stomach and epidermis were collected from E12, and (b) five samples of lung and intestine E14 chick embryos. (c) FOSs workflow (steps 1-4), from harvesting organs to seeding cells in culture flasks. (d) A table indicating the duration of Accutase incubation required to dissociate specific organs into a cell suspension depending on the tissue structure (step 3 in c). Scale bars: 5 mm (white), 2 mm (black).

Figure S2

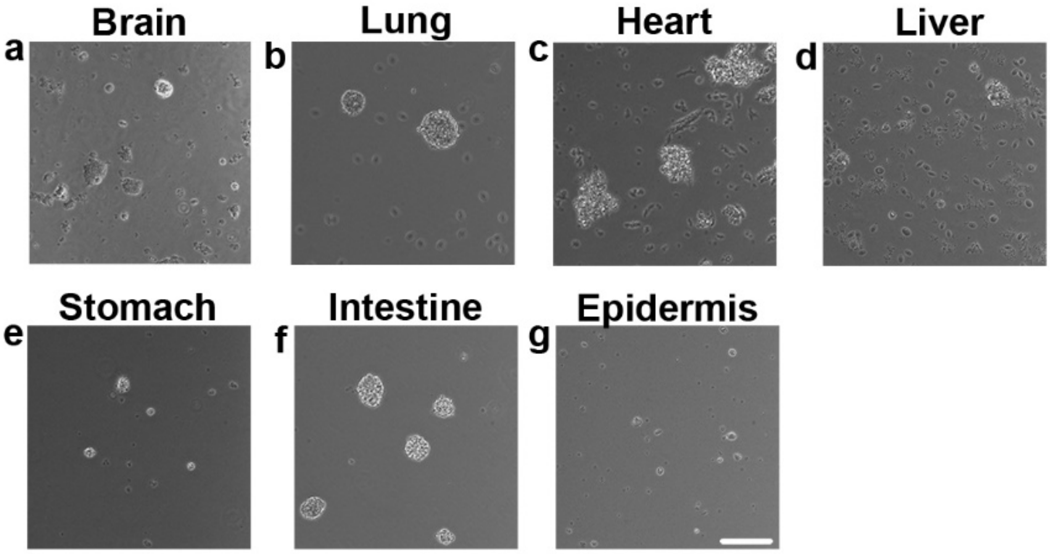

Fig. S2 Day 1 of FOSs cultures. (a-g) Cells from all fetal organs; brain, lung, heart, liver, stomach, intestine and epidermis, formed spheroid-like structures after 1 day in culture. Scale bar: 100 µm.

Figure S3

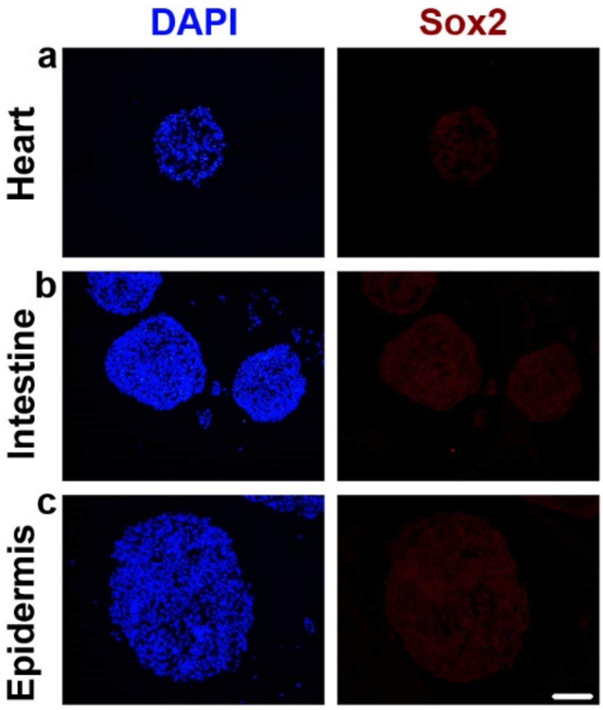

Fig. S3 Sox2 expression analyzed by immunocytochemistry in day 8 in FOSs. (a-c) No expression of Sox2 was detected in day 8 fetal heart, intestinal or epidermal spheroids (n = 7 for all). DAPI indicates nuclei. Scale bar: 100 μm.

Figure S4

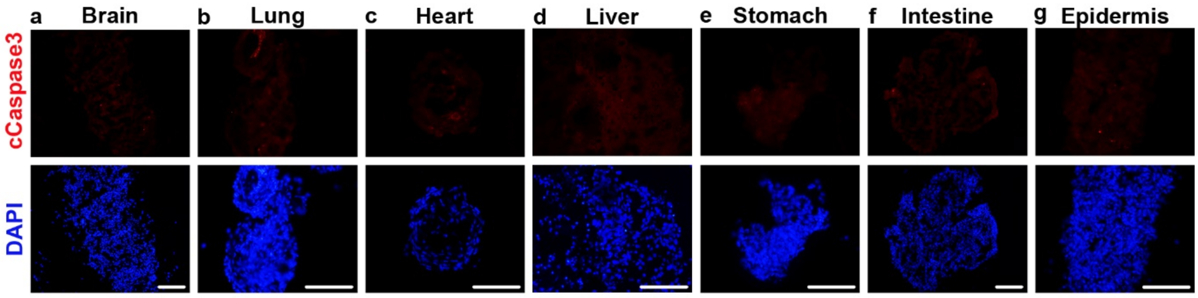

Fig. S4 Expression of cleaved (c) Caspase3 by immunocytochemistry in day 8 FOSs. (a-g) Only a few cCasp3^{+} cells, indicative of apoptotic cells, were observed in FOSs derived from the seven analyzed organs (n = 5 for all FOSs). DAPI indicates nuclei. Scale bar: 100 μm.

Figure S5

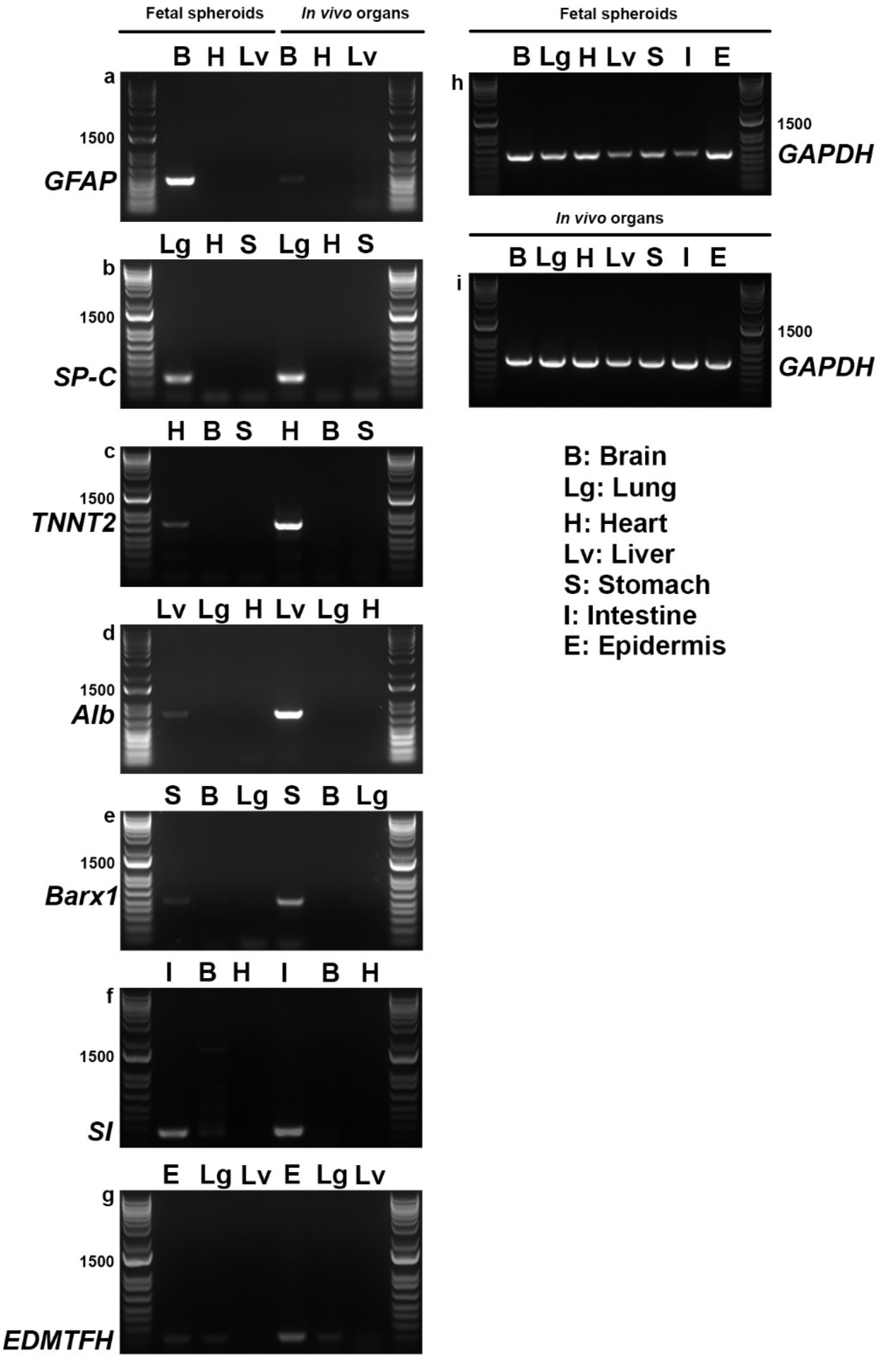

Fig. S5 Full-length gel images of gene expression in seven different FOSs and their corresponding chick embryo *in vivo* organs. (a-i) RT-PCR of day 8 FOSs revealed that mRNA expression of typical organ characteristic genes are enriched in the different FOSs. (a) *GFAP* expression in brain, but not in heart or liver FOSs/organs. (b) *SP-C* expression in lung, but not in heart or stomach FOSs/organs. (c) *TNNT2* expression in heart, but not in brain or stomach

FOSs/organs.(d) *Alb* expression in liver, but not in lung or heart FOSs/organs. (e) *Barx1* expression in stomach, but not in brain or lung FOSs/organs.(f) *SI* expression in intestine, but not in brain or heart FOSs/organs. (g) *EDMTFH* expression in epidermis, but not in lung or liver FOSs/organs. (h, i) The house-keeping gene *GAPDH* was used as a reference gene. (a-i) The first and last lane of each gel image show the ladder, and an amplicon size of 1500 base pairs (bp) are indicated.

Figure S6

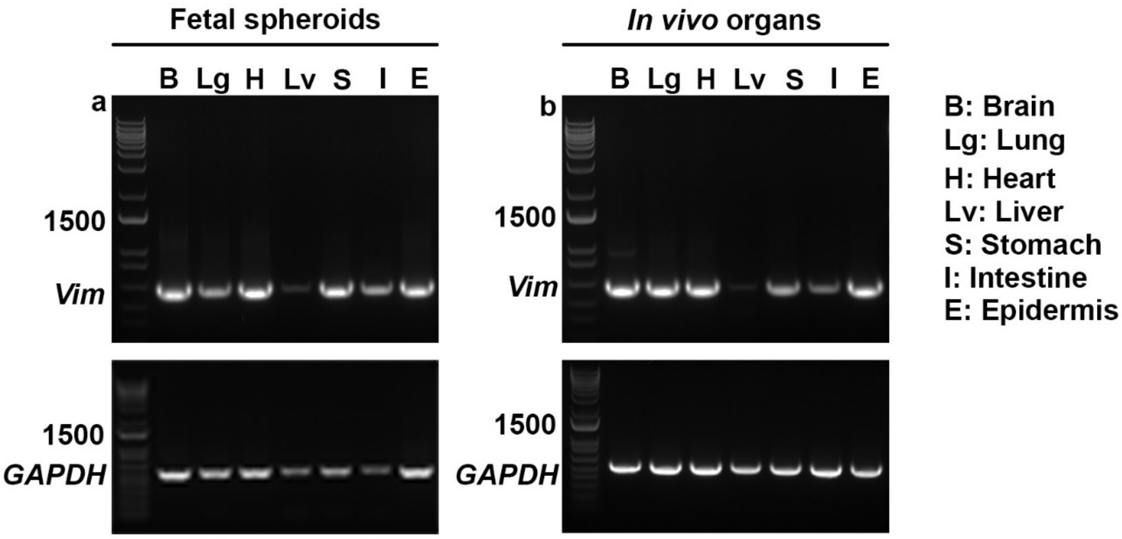

Fig. S6 Full-length gel images of RT-PCR *Vimentin* expression in seven different FOSs and their corresponding chick embryo *in vivo* organs. (a, b) RT-PCR revealed mRNA expression of *Vimentin* (*Vim*) in all day 8 FOSs (a), and in their corresponding embryonic *in vivo* organs (b); E12 brain, heart, liver, stomach and epidermis, and E14 lung and intestine. The house-keeping gene *GAPDH* was used as a reference. The first lane of each gel image shows the ladder, and an amplicon size of 1500 base pairs (bp) is indicated.

Table S1

	D4 size (mm)	D8 size (mm)	D12 size (mm)	Δp		
				D8/D4	D12/D8	D12/D4
Brain	0.66±0.02 [n = 100]	0.95±0.02 [n = 100]	1.88±0.06 [n = 80]	1.43	1.99	2.85
Lung	0.70±0.02 [n = 100]	0.73±0.02 [n = 100]	0.73±0.03 [n = 100]	1.05	1.00	1.04
Heart	0.42±0.02 [n = 80]	0.61±0.04 [n = 75]	0.94±0.03 [n = 100]	1.44	1.54	2.22
Liver	0.74±0.02 [n = 100]	0.82±0.03 [n = 100]	0.98±0.05 [n = 80]	1.11	1.20	1.32
Stomach	1.21±0.06 [n = 100]	1.49±0.06 [n = 75]	1.59±0.08 [n = 76]	1.23	1.07	1.32
Intestine	0.67±0.01 [n = 100]	0.89±0.02 [n = 100]	0.90±0.02 [n = 100]	1.32	1.01	1.33
Epidermis	0.94±0.04 [n = 100]	1.07±0.06 [n = 80]	1.02±0.06 [n = 75]	1.14	0.95	1.09

Table S1. Perimeter of fetal organ spheroids at culture day 4, 8 and 12. All measurements are expressed in mm and stated as mean ± standard error of the mean (SEM). Δp = fold change of the mean perimeter between FOSs measured on the stated days.

Table S2

Gene	Primers	Size (bp)	T (°C)
Glial fibrillary acidic protein (*GFAP*)	F: TGGTACAAGTCCAAGTTT<br>R: TTTTAACCACAATGCTTCT	469	47
Surfactant protein C (*SFTPC* or *SP-C*)	F: CGGTGAAGGAGGGACTCAACGCGAC<br>R: GTGCTGCAGAGGATGTTGATGGTGG	232	55
Cardiac muscle troponin T2 (*TNNT2* or *cTnT*)	F: ATGTGGAAGAAGAAGAGGAAGAATG<br>R: CTCCTGCAAGTCAAATTTCTCAGCC	737	55
Albumin (*Alb*)	F: AACATTAATTTCATTCATTTTCC<br>R: AATTTAGAAACCTCTGAGAA	774	47
Homeobox protein BarH-like 1 *(Barx1*)	F: CACCGCTACCGCAGTTTCATGATTG<br>R: TTTCTTCCATTTCATGCGCCTGTTC	507	55
Sucrase-isomaltase (*SI*)	F: CAACATTTATGGTGTGGGAGAGCATGTG<br>R: GGATGCCGCCAATGGTTCTGTAGG	254	55
Epidermal differentiation protein starting with a MTF motif and rich in histidine (*EDMTFH*)	F: TCTCTGGGGCCTGAATGACCACCGA<br>R: AGTAGGGGTGGCGGTGCCCAAAACC	200	55
Vimentin (*Vim*)	F: CCAGCAGCAAGAACTCCTCGTACCG<br>R: AAGGCGTGCCAGAGAGGCATTGTCA	626	55
Glyceraldehyde-3-phophate dehydrogenase (*GAPDH*)	F: CAATGGGCACGCCATCACTA<br>R: CTCCAGACGGCAGGTCAGGT	544	55

Table S2. Genes detected by RT-PCR. Sequences for each primer pair (forward, F, reverse, R), with indicated amplicon sizes (base pairs, bp) and annealing temperatures (T; ºC).

SUPPLEMENTARY MOVIE CAPTIONS

Movie S1. Video of confocal images of GFP-expressing U251 cancer cells invading the FBSs at day 10 of the confrontation assay (n= 7/7).

Movie S2. Video of confocal images of GFP-expressing U251 cancer cells invading the FBSs at day 10 of the confrontation assay (n= 7/7). DAPI indicates cell nuclei.

Movie S3. Video of confocal images of GFP-expressing U251 cancer cells invading the FLuSs at day 10 of the confrontation assay (n= 4/7).

Movie S4. Video of confocal images of GFP-expressing U251 cancer cells invading the FLuSs at day 10 of the confrontation assay (n= 4/7). DAPI indicates cell nuclei.

Movie S5. Video of confocal images of GFP-expressing A549 cancer cells invading the FBSs at day 10 of the confrontation assay (n= 7/7).

Movie S6. Video of confocal images of GFP-expressing A549 cancer cells invading the FBSs at day 10 of the confrontation assay (n= 7/7). DAPI indicates cell nuclei.

Movie S7. Video of confocal images of GFP-expressing A549 cancer cells invading the FLuSs at day 10 of the confrontation assay (n= 3/8).

Movie S8. Video of confocal images of GFP-expressing A549 cancer cells invading the FLuSs at day 10 of the confrontation assay (n= 3/8). DAPI indicates cell nuclei.
